# Two Nucleolar Proteins, GDP1 and OLI2, Function As Ribosome Biogenesis Factors and Are Preferentially Involved in Promotion of Leaf Cell Proliferation without Strongly Affecting Leaf Adaxial–Abaxial Patterning in *Arabidopsis thaliana*

**DOI:** 10.3389/fpls.2017.02240

**Published:** 2018-01-09

**Authors:** Koji Kojima, Junya Tamura, Hiroto Chiba, Kanae Fukada, Hirokazu Tsukaya, Gorou Horiguchi

**Affiliations:** ^1^Department of Life Science, College of Science, Rikkyo University, Tokyo, Japan; ^2^Graduate School of Science, The University of Tokyo, Tokyo, Japan; ^3^Okazaki Institute for Integrative Bioscience, Okazaki, Japan; ^4^Research Center for Life Science, College of Science, Rikkyo University, Tokyo, Japan

**Keywords:** OLI2, GDP1, ribosome biogenesis, cell proliferation, adaxial–abaxial polarity regulation, leaf development, Arabidopsis

## Abstract

Leaf abaxial–adaxial patterning is dependent on the mutual repression of leaf polarity genes expressed either adaxially or abaxially. In *Arabidopsis thaliana*, this process is strongly affected by mutations in ribosomal protein genes and in ribosome biogenesis genes in a sensitized genetic background, such as *asymmetric leaves2* (*as2*). Most ribosome-related mutants by themselves do not show leaf abaxialization, and one of their typical phenotypes is the formation of pointed rather than rounded leaves. In this study, we characterized two ribosome-related mutants to understand how ribosome biogenesis is linked to several aspects of leaf development. Previously, we isolated *oligocellula2* (*oli2*) which exhibits the pointed-leaf phenotype and has a cell proliferation defect. *OLI2* encodes a homolog of Nop2 in *Saccharomyces cerevisiae*, a ribosome biogenesis factor involved in pre-60S subunit maturation. In this study, we found another pointed-leaf mutant that carries a mutation in a gene encoding an uncharacterized protein with a G-patch domain. Similar to *oli2*, this mutant, named *g-patch domain protein1* (*gdp1*), has a reduced number of leaf cells. In addition, *gdp1 oli2* double mutants showed a strong genetic interaction such that they synergistically impaired cell proliferation in leaves and produced markedly larger cells. On the other hand, they showed additive phenotypes when combined with several known ribosomal protein mutants. Furthermore, these mutants have a defect in pre-rRNA processing. *GDP1* and *OLI2* are strongly expressed in tissues with high cell proliferation activity, and GDP1-GFP and GFP-OLI2 are localized in the nucleolus. These results suggest that OLI2 and GDP1 are involved in ribosome biogenesis. We then examined the effects of *gdp1* and *oli2* on adaxial–abaxial patterning by crossing them with *as2*. Interestingly, neither *gdp1* nor *oli2* strongly enhanced the leaf polarity defect of *as2*. Similar results were obtained with *as2 gdp1 oli2* triple mutants although they showed severe growth defects. These results suggest that the leaf abaxialization phenotype induced by ribosome-related mutations is not merely the result of a general growth defect and that there may be a sensitive process in the ribosome biogenesis pathway that affects adaxial–abaxial patterning when compromised by a mutation.

## Introduction

Ribosome biogenesis drives cellular growth, and, in principle, individual cells must grow twofold before division during the cell cycle. To do so, a large proportion of the gene expression machinery in a cell is devoted to ribosome biogenesis (Warner, 1999). In multicellular organisms, behavior of individual cells is under the control of developmental programs to establish the appropriate shape and function of tissues and organs. Therefore, ribosome biogenesis is expected to be important to developmental pattering. However, the details of this putative connection are not well understood.

In eukaryotes, cytosolic ribosomes consist of a 60S subunit and a 40S subunit. Ribosome biosynthesis initiates in a specialized membraneless nuclear subcompartment, the nucleolus. Pre-ribosomal RNA [pre-rRNA: 45S rRNA in *Arabidopsis thaliana* (Arabidopsis) and 35S rRNA in *Saccharomyces cerevisiae* (yeast)] is transcribed by RNA polymerase I (Pol I) as a polycistronic transcript from rDNA repeats and contains 25–28S, 5.8S, and 18S rRNA sequences that are flanked by a 5′ external transcribed spacer (5′-ETS) and a 3′-ETS and separated by internal transcribed spacer 1 (ITS1) and ITS2 (Layat et al., 2012). Ribosome biogenesis requires more than 200 ribosome biogenesis factors (RBFs) and small nucleolar RNA (snoRNA) species in addition to ribosomal proteins (r-proteins), and has been best characterized in yeast. The 90S pre-ribosome, also designated as the small subunit (SSU) processome, is a huge ribonucleoprotein complex in which a nascent pre-rRNA and a subset of r-proteins for SSU are encapsulated by U three protein (UTP) complexes and U3 small nucleolar ribonucleoprotein (snoRNP). Numerous RBFs dynamically join and dissociate from the 90S pre-ribosome in a hierarchical manner and carry out folding, cleavage, and trimming of rRNA precursors, as well as assembly of r-proteins with rRNAs to produce pre-40S ribosomes (Grandi et al., 2002; Pérez-Fernández et al., 2007; Kornprobst et al., 2016; Chaker-Margot et al., 2017; Sun et al., 2017). The remaining 3′ part of the pre-rRNA forms the large subunit (LSU) processome and produces pre-60S ribosomes (Konikkat and Woolford, 2017). The 5S rRNA is transcribed separately by Pol III and forms a complex with RPL5 and RPL11, then joins to a pre-60S particle (Zhang et al., 2007). Final maturation of pre-ribosomal subunits takes place after export from the nucleus into the cytoplasm (Nerurkar et al., 2015).

Approximately 250 ribosome biogenesis factors have been identified in yeast by genetic and proteomics analyses. Similarly, 286 ribosome biogenesis factors have been identified in human cells, but among them, 74 do not have a yeast ortholog (Tafforeau et al., 2013). For land plants, orthologs to about 75% of yeast ribosome biogenesis factors were identified by bioinformatics methods (Ebersberger et al., 2013). However, only a small fraction of these molecules have been functionally characterized in Arabidopsis (Weis et al., 2015a). These studies suggest that molecular mechanisms for ribosome biogenesis are largely conserved, but that some processes are mediated by species-specific factors. A recent nucleolar proteomics analysis of Arabidopsis supported this interpretation (Palm et al., 2016).

A plant-specific feature of ribosome biogenesis is also seen in two routes of rRNA processing (Weis et al., 2015a). In Arabidopsis, transcription of 45S rRNA is terminated by endonucleolytic cleavage at site B_0_ in the 3′-ETS by RIBONUCLEASE THREE LIKE2 (atRTL2; Comella et al., 2007). Then endonucleolytic cleavage at site P within the 5′-ETS takes place by the action of EXORIBONUCLEASE2 (XRN2) and U3 snoRNP to yield 35S rRNA (Sáez-Vasquez et al., 2004; Zakrzewska-Placzek et al., 2010). There are two alternative routes for further processing of 35S rRNA accompanied by simultaneous removal of the 3′-ETS (Missbach et al., 2013). In one pathway, the 5′-ETS is removed before cleavage within ITS1 (5′-ETS-first pathway) while in the other, cleavage within ITS1 of 35S takes place prior to 5′-ETS cleavage (ITS1-first pathway). In yeast, processing of pre-rRNA strictly follows the 5′-ETS-first pathway, while the ITS1-first pathway is the major route in mammals (Fernández-Pevida et al., 2015; Henras et al., 2015).

In Arabidopsis, impaired function of RBFs affects normal processing of pre-rRNAs as well as diverse developmental processes, such as auxin response, cell proliferation, root epidermal patterning, vascular patterning, leaf shape regulation, callus formation, and development of the gynoecium, embryo, and female gametophyte (Shi et al., 2005; Griffith et al., 2007; Petricka and Nelson, 2007; Lange et al., 2008, 2011; Fujikura et al., 2009; Li et al., 2009; Abbasi et al., 2010; Huang et al., 2010; Liu et al., 2010; Ohbayashi et al., 2011; Wieckowski and Schiefelbein, 2012; Cho et al., 2013; Kumakura et al., 2013; Missbach et al., 2013; Hang et al., 2014; Weis et al., 2014, 2015b). Many of these phenotypes are also shared by mutants defective in a gene for an r-protein (for review, see Byrne, 2009; Horiguchi et al., 2012; Machida et al., 2015). These findings suggest that production and/or function of ribosomes may be associated with developmental regulation. Recently, impaired ribosome biogenesis in mutants, such as *root initiation defective2* (*rid2*), was shown to induce ribosomal stress (Ohbayashi et al., 2017). Explants from *rid2* are unable to form callus at high temperature and produce pointed leaves, but these phenotypes are suppressed by mutations in the NO APICAL MERISTEM, ARABIDOPSIS TRANSCRIPTION ACTIVATION FACTOR1/2 and, CUP-SHAPED COTYLEDON2 (NAC) transcription factor gene *SUPPRESSOR OF RID TWO1* (*SRIW1*)/*ANAC082* (Ohbayashi et al., 2017). Notably, *rid2* mutants are defective in the processing of rRNAs, but this phenotype is not suppressed by *sriw1*, indicating that *SRIW1* mediates ribosomal stress to induce developmental alterations (Ohbayashi et al., 2017).

The potential developmental roles of ribosomes have been further expanded by observations that mutants defective in r-protein significantly enhance the leaf polarity defect of *asymmetric leaves1* (*as1*) and *as2* (Pinon et al., 2008; Yao et al., 2008; Horiguchi et al., 2011; Szakonyi and Byrne, 2011; Casanova-Sáez et al., 2014) and *revoluta* (*rev*) (Pinon et al., 2008). In the double mutants, leaves fail to expand into a flat laminar structure, but form in a trumpet- or needle-like shape. Normally, the vascular tissues are organized such that xylem and phloem tissues face the adaxial (upper) and abaxial (lower) sides of the leaves, respectively (Waites and Hudson, 1995). In contrast, vascular tissues in radialized leaves are organized with the phloem tissues surrounding the vascular tissues, or in extreme cases the vascular tissues are absent (Pinon et al., 2008; Yao et al., 2008; Horiguchi et al., 2011). These double mutants have increased or expanded expression of abaxially expressed genes (Pinon et al., 2008; Yao et al., 2008; Horiguchi et al., 2011), suggesting that mutations in r-protein genes enhance leaf abaxialization in the *as* mutant backgrounds. A similar leaf polarity defect was also found in mutants defective in *Arabidopsis PUMILIO23* (*APUM23*), which encodes a member of the Nop9 family involved in regulation of rRNA processing (Thomson et al., 2007; Abbasi et al., 2010; Droll et al., 2010; Huang et al., 2014; Zhang and Muench, 2015; Zhang et al., 2016). More recently, mutations in *RNA HELICASE10* (*RH10*), *NUCLEOLIN-LIKE1* (*NUC-L1*), and *RID2* were also shown to enhance the leaf polarity defect of *as2* (Matsumura et al., 2016). RH10 is a member of the DEAD-box RNA helicases, putative orthologs of which are Rrp3 in yeast and DDX47 in human (O’Day et al., 1996; Matsumura et al., 2016). NUC-L1 is an ortholog of nucleolin in human and plays multiple roles in ribosome biogenesis (Sáez-Vasquez et al., 2004). RID2 is a homolog of Bud23 in yeast, which is a methyltransferase that catalyzes the methylation of G1575 in 18S rRNA (Ohbayashi et al., 2011; Létoquart et al., 2014). Yeast and human orthologs of RH10, NUC1, and RID2 are components of, or are associated with, the SSU processome (Turner et al., 2009; Phipps et al., 2011; Sardana et al., 2013). These findings imply that correct ribosome production and/or function are required for leaf adaxial/abaxial patterning (Matsumura et al., 2016).

To understand the mechanisms of ribosome biogenesis in plants and how it is connected to developmental programs, we have been trying to genetically identify individual RBFs and have characterized their molecular and developmental phenotypes. Although ribosome biogenesis is highly complicated, comparison of phenotypes among different RBF mutants would provide information concerning the general and specific functions of each factor. The purpose of this study is to find differential requirement of RBFs in two major leaf developmental processes, namely cell proliferation and abaxial–adaxial pattering. In this study, we found a novel gene for a nucleolar protein, G-PATCH DOMAIN PROTEIN1 (GDP1), and characterized it together with previously identified OLIGOCELLULA2 (OLI2; Fujikura et al., 2009), which is a homolog of yeast nucleolar protein Nop2 involved in formation of 5-methylcytosine (m5C) at C2870 in 25S rRNA (Sharma et al., 2013). Loss-of-function mutants of *GDP1* and *OLI2* exhibited a range of phenotypes frequently found in mutants defective in r-proteins or RBFs. Interestingly, *gdp1, oli2*, and double mutants between them did not show strongly enhanced *as2* leaf polarity defects despite their association with significantly impaired cell proliferation in leaves. These results suggest that the roles of ribosome biogenesis/function in leaf adaxial/abaxial patterning and other developmental processes are at least partially separate.

## Materials and Methods

### Plant Materials

The wild type accession of Arabidopsis used in this study was Columbia-0 (Col-0). T-DNA insertion lines (Salk_065904 [*gdp1-1*], Salk_041661 [*gdp1-2*]) were obtained from the Arabidopsis Biological Resource Center (ABRC). *gdp1-3* was previously identified as #416 (Horiguchi et al., 2006a,b), and *oli2-1* and *oli2-2* (Salk_129648) were reported previously (Fujikura et al., 2009). Seeds were sown on rockwool and grown at 22°C under a 16-h light/8-h dark photoperiod for quantitative characterization of leaf phenotypes and RNA preparation. Seedlings were watered daily with 0.5 g L^-1^ of Hyponex (Hyponex Japan). For fluorescence imaging of root, seedlings were grown for 5 days on half-strength Murashige and Skoog (MS) medium supplemented with Gamborg B5 vitamins and 3% (w/v) sucrose, and solidified with 0.5% (w/v) gellan gum at 22°C under a 16-h light/8-h dark photoperiod. Oligonucleotide pairs for genotyping of *gdp1* alleles are listed in Supplementary Table S1.

### Generation of Transgenic Plants

An approximately 1.6-kb *GDP1* promoter region with or without the *GDP1* transcribed region was amplified and cloned into pDONR201 using BP clonase (Thermo) followed by transfer of the insert into pBGGUS (Horiguchi et al., 2005) or pHWG (Horiguchi and Tsukaya, unpublished) with LR clonase II (Thermo) to yield p*GDP1*::*GUS* and p*GDP1*::*GDP1*-*GFP* constructs, respectively. For construction of p*OLI2*::*GUS*, an *OLI2* promoter DNA fragment and *GUS* cDNA were cloned into pDONR P4P1R and pENTR/D-TOPO (Thermo), respectively, and combined into the Gateway binary vector, pGWB501 (Nakagawa et al., 2007), with LR clonase II plus (Thermo). For construction of p*OLI2*::*GFP*-*OLI2*, a 3.6-kb promoter region of *OLI2, GFP* cDNA, and a transcribed region plus a 4.1-kb 3′ untranscribed region of *OLI2* were amplified and cloned into pSMAH621 digested with HindIII and SacI using an In-Fusion HD cloning kit (Clontech). These constructs were introduced into Arabidopsis by the floral dip method (Clough and Bent, 1998). Transgenic plants with a single T-DNA insertion were selected and homozygous T3 plants were used. Oligonucleotides used in the construction of these vectors are listed in Supplementary Table S1.

### RNA Analyses

Total RNA was prepared from shoots of wild type, *gdp1, oli2*, and *gdp1 oli2* using Trizol reagent (Thermo). Isolated RNA was treated with DNase I (Thermo) followed by first-strand cDNA synthesis with SuperScript III reverse transcriptase (Thermo) primed either with oligo (dT) (for messenger RNA) or random hexamers (for rRNAs). The cDNAs were subjected to semi-quantitative and quantitative RT-PCR (RT-qPCR). RT-qPCR was carried out using GoTaq qPCR master mix (Promega) with an ABI7500 real-time PCR system (Thermo) by the ΔΔ*C*t method. The expression level of *ACTIN2* (*ACT2*) was used as an endogenous control for mRNAs, while 18S rRNA was used as an endogenous control to determine rRNA intermediate levels. Oligonucleotide pairs used to detect *AUXIN RESPONSE FACTOR3* (*ARF3*), *KANADI2* (*KAN2*), *YABBY5* (*YAB5*), *PHABOLUSA* (*PHB*), *PHAVOLUTA* (*PHV*), *REV*, and *ACT2* were described previously (Iwakawa et al., 2007). Other oligonucleotide pairs are listed in Supplementary Table S1.

For detection of rRNA intermediates by Northern hybridization, aliquots of 3 μg of total RNAs isolated from 12-day-old shoots were separated by formaldehyde-agarose gel electrophoresis (1.2%, w/v) and transferred onto nylon membranes by downward capillary transfer. After ultraviolet crosslinking of RNA to the nylon membranes, hybridization was carried out using a DIG Northern starter kit (Sigma–Aldrich) and hybridization signals were detected using a digital imaging system (LAS 4000 mini; GE Healthcare). RNA probes were generated using 5′-ETS, ITS1, and ITS2 DNA fragments amplified with the appropriate oligonucleotide pairs (Supplementary Table S1) and T7 RNA polymerase.

### Microscopic Observation

Quantitative analyses of leaves were carried out as described (Horiguchi et al., 2005). Plants were gron for 25 days and first leaves were fixed in a formalin/acetic acid/alcohol [FAA, formalin: acetic acid: 70% (v/v) ethanol = 1:1:18] solution, cleared using a chloral hydrate solution (200 g chloral hydrate, 20 g glycerol, and 50 ml dH_2_O) and observed by stereomicroscope (M165FC; Leica) and differential contrast interference microscope (DM2500; Leica), respectively. Leaf blade area and the projection area of palisade cells in adaxial subepidermal layer was determined using Image J^1^. For each leaf, the palisade cell area was determined by mearuing at least 20 cells. Average palisade cell size was determined using data from 10 leaves. Cell density in adaxial subepidermal layer was manuary determined by counting cells in a unit area. The number of cells in adaxial subepidermal layer per leaf was estimated by dividing the leaf blade area with the cell density. For fluorescence imaging of p*OLI2*::*GFP*-*OLI2*/*oli2-1* and p*GDP1*::*GDP1*-*GFP*/*gdp1-1* lines, roots of 5-day-old seedlings were fixed in phosphate buffered saline (PBS, pH 7.0) containing 4% (w/v) paraformaldehyde, washed twice with PBS, and stained with 4′,6-diamidine-2′-phenylindole dihydrochloride (DAPI; Merck) or Calcofluor White M2R (Merck) and cleared with TOMEI-II (Hasegawa et al., 2016). For fluorescence imaging of leaves, 14-day-old shoots grown on rock wool were fixed in PBS [pH 7.0 containing 4% (w/v) paraformaldehyde, washed twice with PBS, and treated with TOMEI-II]. Fluorescent signals were observed with a confocal laser scanning microscope (LSM710 or LSM800; Zeiss). Histochemical staining of promoter::*GUS* lines was carried out according to Donnelly et al. (1999).

## Results

### Identification of *gdp1* Mutants and Characterization of Their Vegetative Phenotypes

**FIGURE 1:**
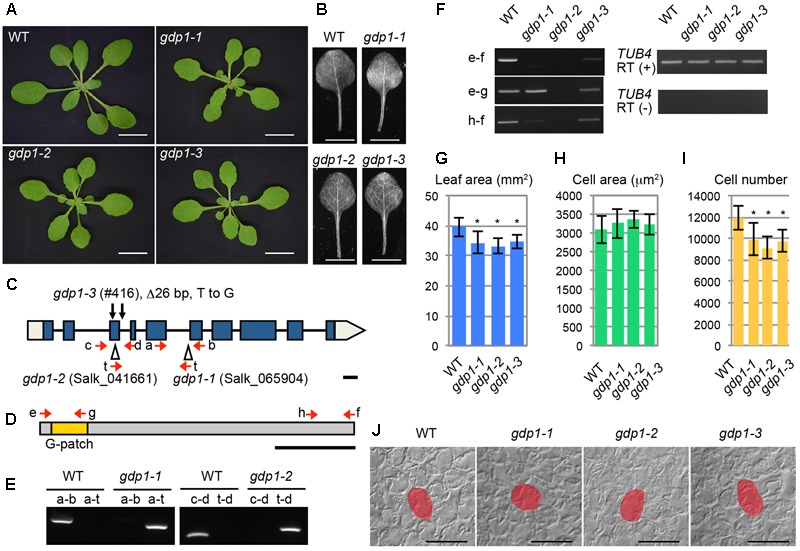
Genetic analyses of *gdp1* alleles. **(A)** Shoots of wild type (WT) and *gdp1-1, gdp1-2*, and *gdp1-3* alleles grown for 25 days. Bars, 1 cm. **(B)** The first leaves of WT and *gdp1-1, gdp1-2*, and *gdp1-3* alleles. Bar, 5 mm. **(C)** Schematic diagram of mutation points of *gdp1* alleles. Red arrows indicate oligonucleotides used for genotyping of *gdp1* alleles. Black arrows indicate the mutation points in *gdp1-3*. T-DNA insertions in *gdp1-1* and *gdp1-2* are indicated by triangles. Bar, 100 bp. **(D)** Schematic diagram of GDP1 protein. Arrows indicate the positions of oligonucleotides on the corresponding *GDP1* transcript used for RT-PCR analysis. Bar, 100 amino acid residues. **(E)** Genotyping of *gdp1-1* and *gdp1-2* alleles. Oligonucleotide pairs indicated by letters were used to amplify genomic DNA fragments from the WT and the two *gdp1* alleles. **(F)** RT-PCR analysis of *GDP1* transcripts. Total RNAs were isolated from 10-day-old shoots and subjected to reverse transcription followed by PCR with oligonucleotide pairs indicated by letters. **(G–I)** Quantitative analyses of leaf phenotypes. The leaf blade area **(G)**, the projection area of leaf mesophyll cells **(H)**, and the number of leaf mesophyll cells **(I)** in the adaxial subepidermal layer of palisade tissues of first leaves are shown. First leaves were harvested from 25-day-old plants. *n* = 10, mean ± SD. Asterisks indicate statistically significant differences compared to WT (*P* < 0.05, Student’s *t*-test). **(J)** Leaf palisade cells observed from a paradermal view. Bar, 100 μm. Representative cells are highlighted.

During the course of studying mutants with altered leaf size and shape, we found that two T-DNA insertion lines of At1g63980 (Salk_065904 and Salk_041661) exhibited the “pointed-leaves” phenotype that is typically observed in mutants defective in r-protein genes and ribosome biogenesis genes (**Figures 1A–C,E**). An additional allele was also found in our mutant collection reported previously (#416; Horiguchi et al., 2006a,b) that had a 26-bp deletion in the third exon and a point mutation in the third intron (**Figures 1A–C**). As At1g63980 was not a characterized gene and it was apparent that At1g63980 does not encode an r-protein, we decided to characterize these mutants in relation to ribosome biogenesis.

The At1g63980 gene product has a G-patch domain near its amino-terminal region (**Figure 1D**). The G-patch domain is about 48 amino acid residues in length and contains several conserved glycine residues (Supplementary Figure S1). This domain is often found in RNA processing proteins with or without known RNA-binding motifs (Aravind and Koonin, 1999). Therefore, we named At1g63980 *G-PATCH DOMAIN PROTEIN1* (*GDP1*), and Salk_065904, Salk_041661, and #416 were designated as *gdp1-1, gdp1-2*, and *gdp1-3*, respectively (**Figure 1C**). The Arabidopsis genome includes at least 15 G-patch domain-containing protein genes. The structures of individual G-patch domain proteins differ in the location and number of G-patch domains, combination of RNA-binding motifs, and amino acid lengths (Supplementary Figure S1). Among them, *GDP1* appears to be a single-copy gene.

The two T-DNA insertion alleles of *gdp1* did not accumulate *GDP1* transcripts at a detectable level, while *gdp1-3* showed *GDP1* transcript accumulation at a lower level than wild type, as determined by RT-PCR using an oligonucleotide pair that amplifies the whole coding region (**Figure 1F**). When oligonucleotide pairs designed to amplify partial *GDP1* cDNA fragments corresponding to the 5′ and 3′ regions of the coding sequence were used, the 5′ fragment was amplified in *gdp1-1* at a level similar to the wild type, but neither the 5′ nor 3′ regions were detectable in *gdp1-2* (**Figure 1F**). In *gdp1-3*, both fragments were detected at lower levels than in wild type (**Figure 1F**). These results suggest that *gdp1-2* is a null allele. We also considered both *gdp1-1* and *gdp1-3* to be strong loss-of-function alleles as their phenotypes were almost identical to *gdp1-2*, as shown below.

**FIGURE 2:**
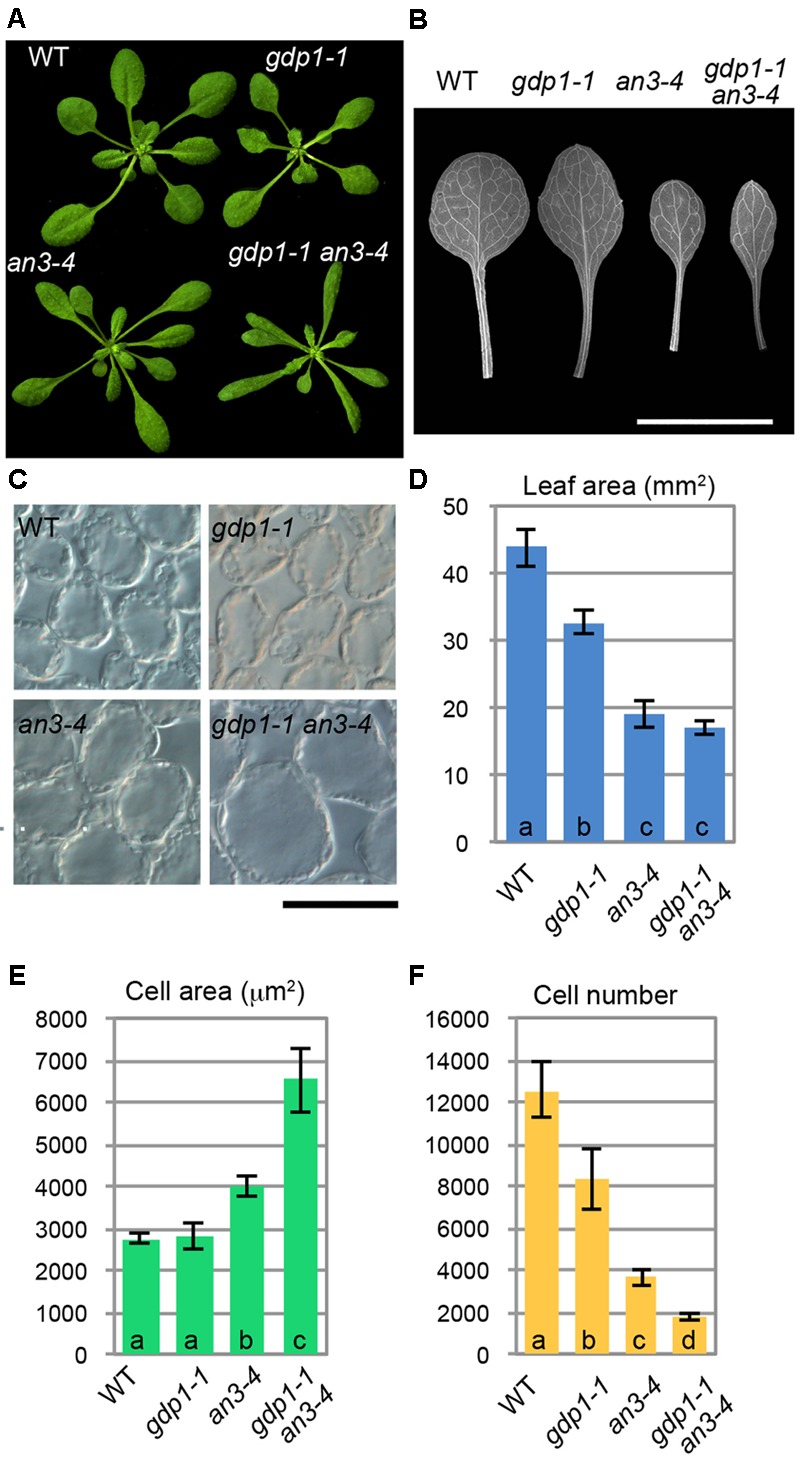
Genetic interaction between *gdp1* and *an3*. **(A)** Shoots of 25-day-old WT, *gdp1-1, an3-4*, and *gdp1-1 an3-4*. **(B)** The first leaves of WT, *gdp1-1, an3-4*, and *gdp1-1 an3-4*. Bar, 1 cm. **(C)** Leaf palisade cells observed from a paradermal view. Bar, 100 μm. **(D–F)** Quantitative analyses of leaf phenotypes. Leaf blade area **(D)**, the projection area of leaf mesophyll cells **(E)**, and the number of leaf mesophyll cells **(F)** in the adaxial subepidermal layer of palisade tissues of first leaves are shown. First leaves were harvested from 25-day-old plants. *n* = 10, mean ± SD. Statistically significant differences were indicated by different letters (one-way ANOVA with Tukey HSD test, *p* < 0.05).

We examined the leaf phenotypes of *gdp1* at the cellular level. Both the area of the leaf blade and the number of palisade cells in the subepidermal layer of the first leaves were reduced by about 20% in *gdp1* compared to the wild type (**Figures 1G,I**). On the other hand, the projection area of palisade cells was similar in both *gdp1* and wild type (**Figures 1H,J**). These phenotypes were similar to those observed in *oli2, oli7*, and *oli5*, the latter two of which are defective in paralogous r-protein genes, *RPL5A* and *RPL5B* (Fujikura et al., 2009). These *oli* mutants showed strongly enhanced cell enlargement in leaves of *angustifolia3* (*an3*), which is defective in a transcription coactivator (Horiguchi et al., 2005) by further reducing the leaf cell number (Fujikura et al., 2009). Similar to these mutants, when *gpd1-1* was crossed with *an3-4*, the resultant double mutant showed a further decrease in number of leaf palisade cells in the subepidermal layer (86% fewer than wild type) and triggered excessive cell enlargement (**Figure 2**; 237% larger than wild type), which is known as compensated cell enlargement (Horiguchi and Tsukaya, 2011; Hisanaga et al., 2015).

**FIGURE 3:**
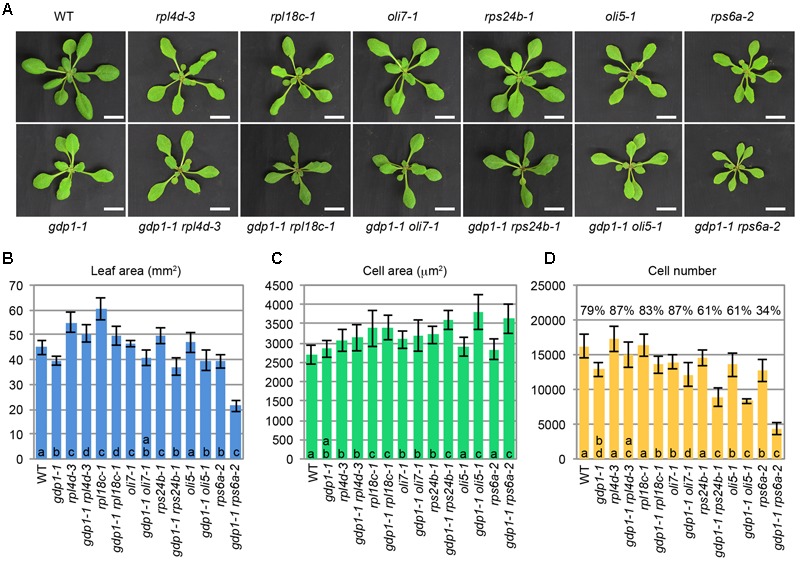
Genetic interactions between *gdp1* and various r-protein mutants. **(A)** Shoots of 25-day-old WT, *gdp1*, r-protein mutants, and double mutants between them. Bar, 1 cm. **(B–D)** Quantitative analyses of leaf phenotypes. Leaf blade area **(B)**, the projection area of leaf mesophyll cells **(C)**, and the number of leaf mesophyll cells **(D)** in the adaxial subepidermal layer of palisade tissues of first leaves are shown. First leaves were harvested from 25-day-old plants. *n* = 10, mean ± SD. Statistically significant differences among four plant lines namely, wild type, *gdp1-1*, single r-protein mutants, and double mutants between *gdp1* and the r-protein mutants were indicated by different letters (one-way ANOVA with Tukey HSD test, *p* < 0.05). In **(D)**, relative cell number in *gdp1* and double mutants on the basis of WT and parental single r-protein mutants, respectively, are shown.

The above developmental phenotypes are quite similar to those observed in mutants defective in r-protein genes and ribosome biosynthesis genes. To examine potential genetic interactions between *gdp1* and these ribosome-related mutants, *gdp1-1* was crossed with various r-protein defective mutants reported previously (**Figure 3**; Horiguchi et al., 2011). Generally, these double mutants showed an additive phenotype as determined from the number of leaf palisade cells (**Figure 3D**). The double mutants showed reduction of leaf palisade cells in the subepidermal layer by 13–39% when compared with respective parental r-protein mutants (**Figure 3D**). In relation to the projection area of palisade cells, the double mutants tended to have a larger cell size than parental r-protein mutants, showing the occurrence of compensated cell enlargement (**Figure 3C**). Consequently, the overall shoot size and first leaf size were less significantly reduced from single r-protein mutants (**Figures 3A,B**). A unique exception was found in *rps6a-2*. Strikingly, *gdp1-1 rps6a-2* had 66% fewer leaf palisade cells in the subepidermal layer when compared with *rps6a-2*, suggesting a synergistic interaction between these two mutations (**Figure 3D**).

**FIGURE 4:**
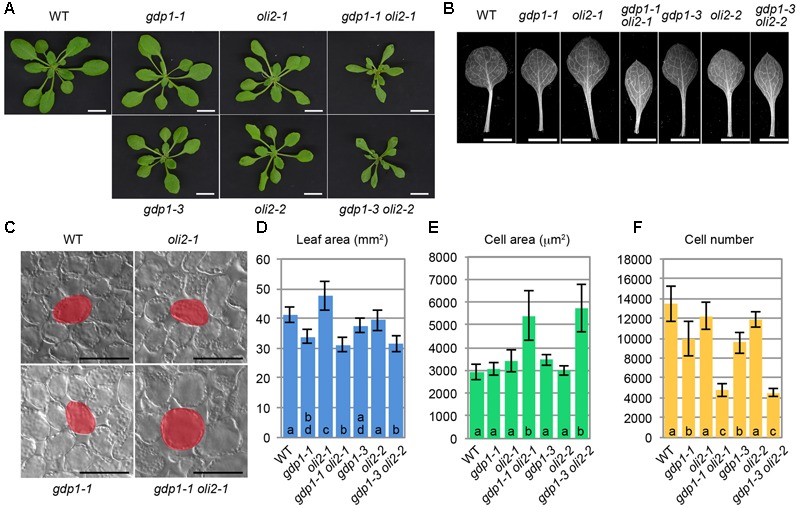
Genetic interaction between *gdp1* and *oli2*. **(A)** Shoots of 25-day-old WT, *gdp1* alleles, *oli2* alleles, and double mutants between them. Bars, 1 cm. **(B)** First leaves. Bars, 5 mm. **(C)** Leaf palisade cells observed from a paradermal view. Bar, 100 μm. **(D–F)** Quantitative analyses of leaf phenotypes. Leaf blade area **(D)**, the projection area of leaf mesophyll cells **(E)**, and the number of leaf mesophyll cells **(F)** in the adaxial subepidermal layer of palisade tissues of first leaves are shown. First leaves were harvested from 25-day-old plants. *n* = 10, mean ± SD. Statistically significant differences were indicated by different letters (one-way ANOVA with Tukey HSD test, *p* < 0.05).

We also examined genetic interactions between *gdp1-1* and *oli2-1* (**Figure 4**). The double mutants between *gdp1-1* and *oli2-1* produced smaller shoots and narrower leaves than the respective parental mutants (**Figures 4A,B,D**). The double mutants frequently produced monocots or tricots that were rarely observed in the single mutants (Supplementary Figure S2). Although the effect of each single mutation on the number of leaf palisade cells in the subepidermal layer was relatively weak (only 10–30% reduction), the double mutants showed a reduction by about 65% compared to the wild type level (**Figure 4F**). The projection area of leaf palisade cells was more than 83% larger in *gdp1-1 oli2-1* than the wild type, indicating compensated cell enlargement (**Figures 4C,E**). These phenotypes were found in different allelic combinations, *gdp1-3 oli2-2* (**Figure 4**). As *OLI2* encodes a putative m5C methyltransferase and likely participates in ribosome biogenesis (Fujikura et al., 2009), the strong genetic interaction between *gdp1* and *oli2* suggests a role of GDP1 in ribosome biogenesis.

### Tissue-Specific *GDP1* and *OLI2* Expression and Subcellular Localization of Their Gene Products

**FIGURE 5:**
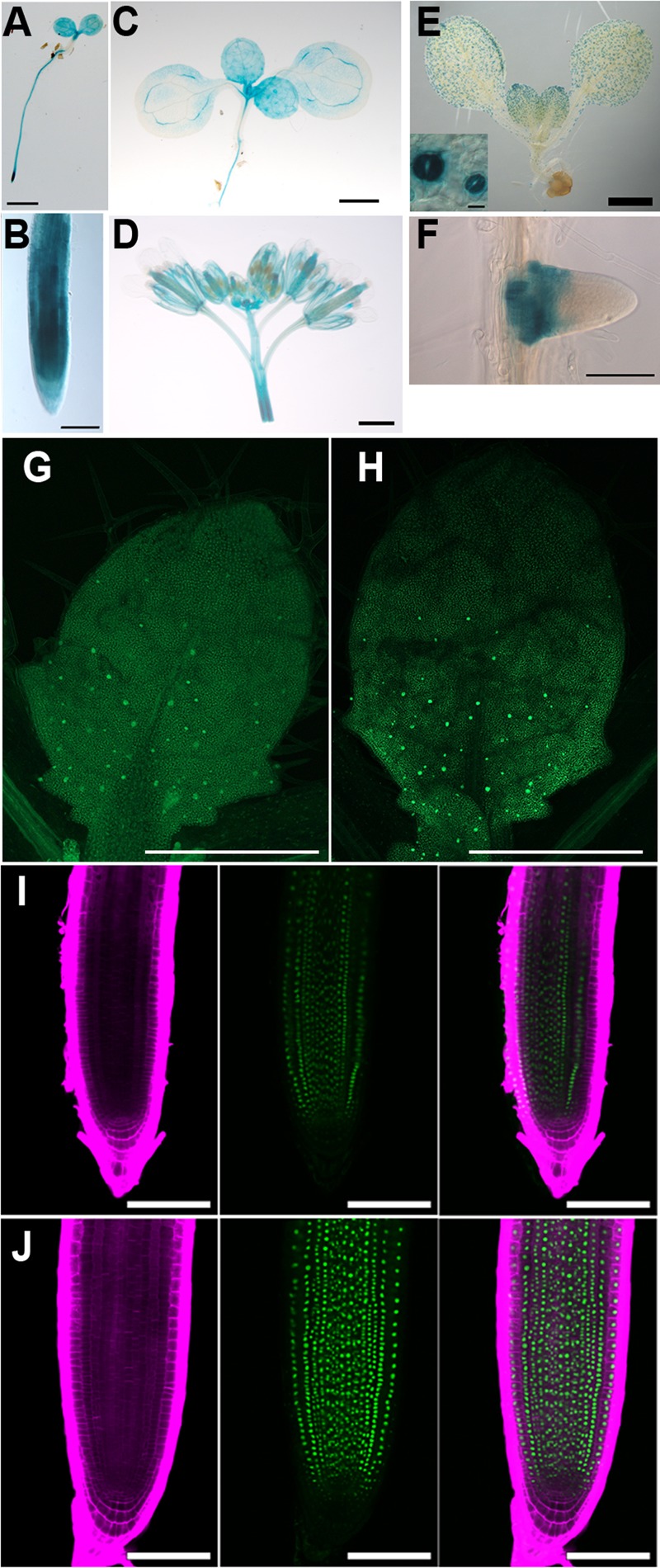
Tissue-specific expression analysis of *GDP1* and *OLI2*. **(A–D)** Histochemical staining of p*GDP1*::*GUS* plants. **(A)** A 4-day-old seedling. **(B)** Primary root tip. **(C)** A 12-day-old seedling. **(D)** Inflorescence. **(E,F)** Histochemical staining of p*OLI2*::*GUS* plants. **(E)** A 5-day-old seedling. The insert shows a close-up view of guard cells in a cotyledon. **(F)** A lateral root. **(G,H)** Confocal images of 5-day-old p*GDP1*::*GDP1-GFP*/*gdp1-1* **(G,I)** and p*OLI2*::*GFP-OLI2*/*oli2-1* **(H,J)**. **(G,H)** Leaf primordia. **(I,J)** Primary root tips. Calcofluor fluorescence, GFP fluorescence, and merged images are shown from left to right. Bars in **(A,C–E)**: 1 mm, **(B,F,I,J)**: 100 μm, insert in **(E)**: 50 μm, **(G,H)**: 0.5 mm.

**FIGURE 6:**
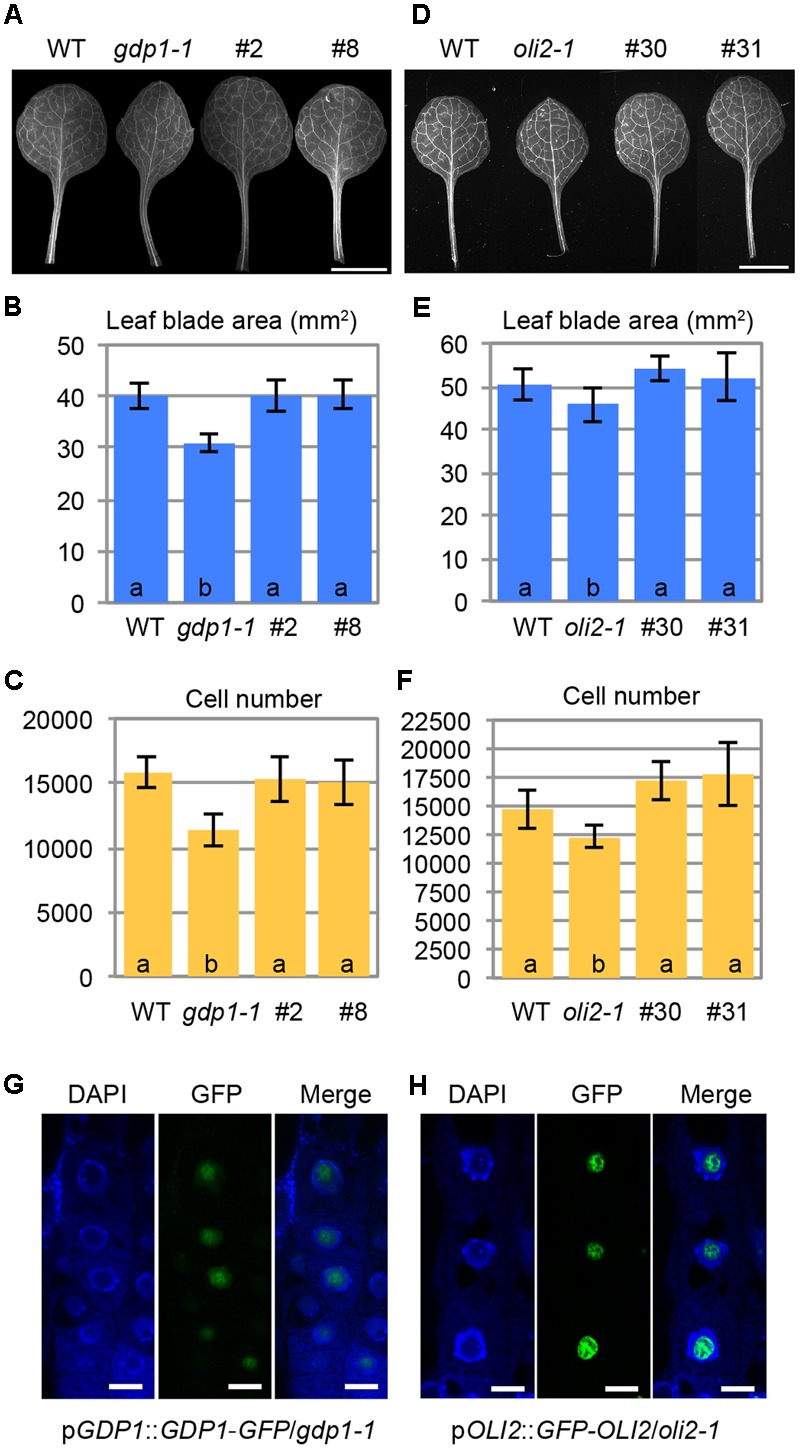
Intracellular localization of GDP1-GFP and GFP-OLI2. **(A,D)** First leaves of transgenic *gdp1-1* lines harboring a p*GDP1*::*GDP1*-*GFP* construct (#2 and #8) **(A)** and a p*OLI2*::*GFP*-*OLI2* construct (#30 and #31) **(D)** grown for 25 days. **(B,C,E,F)** Quantitative analyses of leaf phenotypes of the *gdp1* **(B,C)** and *oli2* **(E,F)** transgenic lines. Leaf blade area **(B,E)**, and the number of leaf mesophyll cells **(C,F)** in the adaxial subepidermal layer of palisade tissues of first leaves are shown. In **(B,C,E,F)**, data are means ± SD (*n* = 10). Statistically significant differences were indicated by different letters (one-way ANOVA with Tukey HSD test, *p* < 0.05). **(G,H)** Subcellular localization of GDP1-GFP **(G)** and GFP-OLI2 **(H)** in cells stained with DAPI. Fluorescence images of DAPI, GFP, and merged images are shown from left to right. Bars, 10 μm.

When *GDP1* expression was observed in a p*GDP1*::*GUS* transgenic line, relatively strong GUS staining was detected in the shoot tip, young leaf primordium, root tip, and floral buds (**Figures 5A–D**). Active ribosome biogenesis takes place in the proliferating cell population. Therefore, *GDP1* expression in actively developing tissues is consistent with the expected function of GDP1 in ribosome biogenesis. On the other hand, p*OLI2*::*GUS* transgenic lines showed GUS staining in guard cells and the basal parts of lateral roots rather than in these tissues (**Figures 5E,F**). We considered this result to indicate that the promoter region used in the transgenic line was insufficient to show the authentic expression pattern of *OLI2*. To overcome this problem, we generated p*OLI2*::*GFP*-*OLI2*/*oli2-1* lines. These transgenic lines fully complemented the leaf shape of *oli2-1* (**Figures 6D–F**). In relation to cell proliferation, the two p*OLI2*::*GFP*-*OLI2*/*oli2-1* lines had even greater numbers of leaf palisade cells in the subepidermal layer than the wild type (**Figure 6F**). In contrast to the p*OLI2*::*GUS* lines, strong GFP-OLI2 signals were observed in leaf primordia and root apical meristem (**Figures 5H,J**). We also generated two p*GDP1*::*GDP1*-*GFP*/*gdp1-1* lines that also complemented the *gdp1* leaf phenotypes (**Figures 6A–C**). GDP1-GFP signals were also found in root tips and leaf primordia (**Figures 5G,I**). In addition, strong expression levels of *GDP1* and *OLI2* were found using the electronic Fluorescent Pictograph (eFP) Browser (Winter et al., 2007). These results suggest that both *GDP1* and *OLI2* are strongly expressed in actively growing tissues that have a high demand for ribosome production.

No information is available regarding the subcellular localization of GDP1 even in nucleolar proteomics analyses (Brown et al., 2005; Pendle et al., 2005; Palm et al., 2016), while OLI2 was detected as a nuclear/nucleolar protein (Palm et al., 2016). We examined the subcellular localization of GDP1-GFP and GFP-OLI2 in root tips stained with DAPI. DAPI stains nuclear chromosomal DNA, and the nucleolus is recognized as a round and dark region in the center of the nucleus. Both GDP1-GFP and GFP-OLI2 signals were found in DAPI-negative nuclear regions (**Figures 6G,H**). As the nucleolus is the center of ribosome biogenesis, the nucleolar localization of GDP1 and OLI2 further supported their roles in ribosome biogenesis.

### Processing of rRNAs in *gdp1, oli2*, and *gdp1 oli2*

**FIGURE 7:**
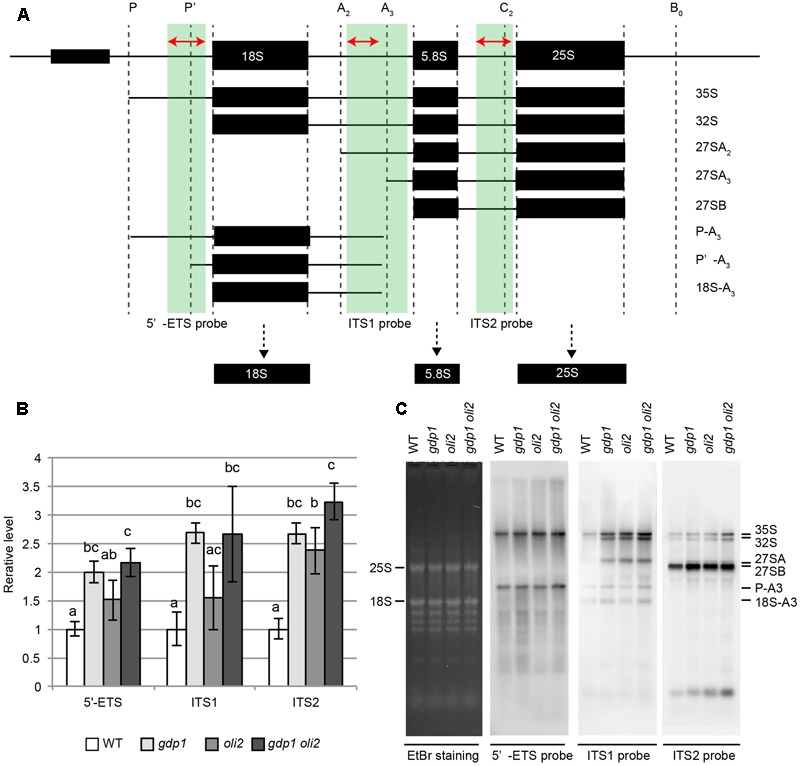
Accumulation of rRNA intermediates. **(A)** Brief overview of rRNA intermediates detected by either RT-qPCR or Northern hybridization. Parts of rRNA intermediates amplified by RT-qPCR and those detected by Northern hybridization are indicated by red arrows and green boxes, respectively. **(B)** RT-qPCR analysis of rRNA intermediates. *n* = 3, mean ± SD. Statistically significant differences were indicated by different letters (one-way ANOVA with Tukey HSD test, *p* < 0.05). **(C)** Northern hybridization of total RNAs. Shoots of 12-day-old WT, *gdp1-1, oli2-1*, and *gdp1-1 oli2-1* were used.

We next examined the effects of *gdp1* and *oli2* mutations on rRNA processing. A brief overview of rRNA processing intermediates relevant to this experiment is shown in **Figure 7A**. We first examined whether the levels of rRNA intermediates were altered in these mutants. RT-qPCR analysis suggested that rRNA intermediates containing 5′-ETS, ITS1, or ITS2 accumulated at higher levels in *gdp1-1, oli2-1*, and *gdp1-1 oli2-1* than in the wild type (**Figure 7B**). The *oli2-1* mutation seemed to have a stronger negative effect on rRNA processing of ITS2-containing intermediates than those containing 5′-ETS or ITS1. On the other hand, *gdp1-1* had a broader impact on the accumulation of rRNA intermediates compared to *oli2-1* as the levels of 5′-ETS-, ITS1-, or ITS2-containing rRNA intermediates accumulated by more than twofold compared to the wild type. Unexpectedly, the 5′-ETS-, ITS1-, and ITS2-containing intermediates accumulated at similar levels in *gdp1-1 oli2-1* compared to those found in *gdp1-1*, despite their synergistic negative effect on cell proliferation (**Figure 7B**). Next we examined the patterns of rRNA intermediate accumulation by Northern hybridization (**Figure 7C**). In *gdp1-1, oli2-1*, and *gdp1-1 oli2-1*, 35S rRNA accumulated at higher levels than in the wild type. These mutants also accumulated 27SA, 27SB, P-A3, and 18SA3 rRNAs at higher levels than those seen in the wild type. Similar to the results of RT-qPCR analyses (**Figure 7B**), *gdp1-1 oli2-1* showed only modest increases, if any, in levels of these rRNA intermediates compared to their parental mutants (**Figure 7C**). These results indicate that OLI2 and GDP1 are required for normal progression of rRNA processing.

### Effects of *gdp1* and *oli2* on the Leaf Polarity Defects of *as2*

**FIGURE 8:**
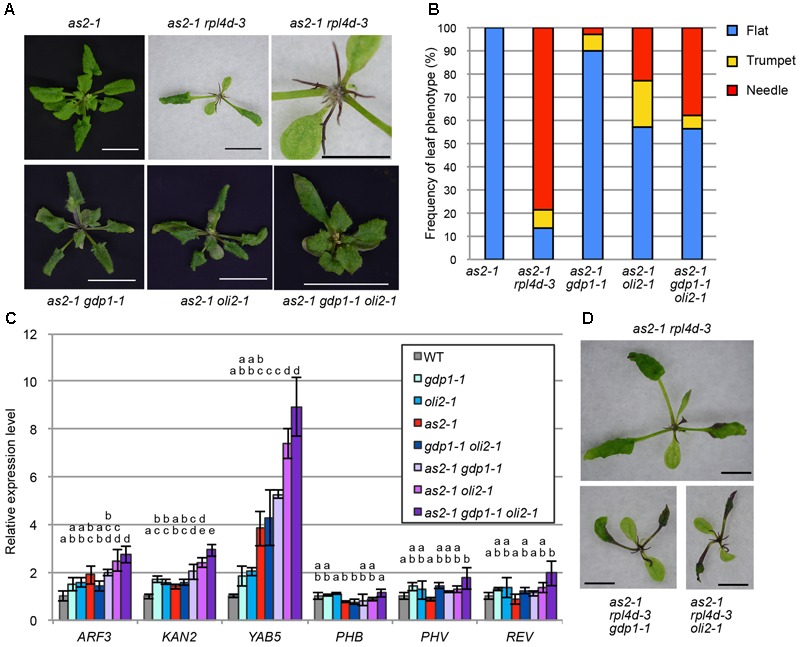
The effects of *gdp1* and *oli2* on leaf development in the *as2* background. **(A)** Shoots of *as2-1, as2-1 rpl4d-3, as2-1 gdp1-1, as2-1 oli2-1*, and *as2-1 gdp1-1 oli2-1* grown for 25 days. Bars indicate 1 cm except for the close-up view of *as2-1 rpl4d-3* where it shows 5 mm. **(B)** Frequencies of flat, trumpet-like, and needle-like leaves. More than 13 plants were examined and all rosette and cauline leaves were scored. **(C)** Expression levels of leaf polarity genes. Shoots of 10-day-old seedlings were used for RNA extraction. *n* = 3, mean ± SD. **(D)** Shoots of *as2-1 rpl4d-3, as2 rpl4d-3 gdp1-1*, and *as2 rpl4d-3 oli2-1* grown for 25 days. Bars, 1 mm. Statistically significant differences were indicated by different letters (one-way ANOVA with Tukey HSD test, *p* < 0.05).

As many mutants defective in an r-protein gene show the enhanced leaf abaxialization phenotype of *as2*, we examined whether *gdp1* and *oli2* also have similar developmental effects. In this experiment, we included *as2-1 rpl4d-3*, which was reported previously to show very strong leaf abaxialization (Horiguchi et al., 2011). The *as2-1 oli2-1* plants showed only moderately enhanced leaf abaxialization, as determined from the frequencies of formation of needle and trumpet-like leaves (**Figures 8A,B**). On the other hand, *gdp1-1* mutation had an even weaker effect on the leaf polarity defect of *as2* than *oli2-1*; *as2-1 gdp1-1* only occasionally produced trumpet- and needle-like leaves (**Figures 8A,B**). We also generated *as2-1 gdp1-1 oli2-1* triple mutants. In contrast to the synergistic negative effect of *gdp1-1* and *oli2-1* on cell proliferation (**Figure 4**), the triple mutant showed only slight enhancement of the leaf polarity defect compared to *as2-1 oli2-1* (**Figures 8A,B**). Next, we examined the expression levels of leaf polarity genes. Abaxially expressed genes, such as *ARF3, KAN2*, and *YAB5*, were slightly upregulated in *gdp1-1, oli2-1*, and *as2-1*, and the degree of upregulation increased progressively in double mutant combinations among *as2-1, gdp1-1*, and *oli2-1* and *as2-1 gdp1-1 oli2-1* triple mutants (**Figure 8C**). On the other hand, the expression levels of adaxially expressed genes, such as *PHB, PHV*, and *REV*, were relatively constant between wild type and both single and multiple mutants examined (**Figure 8C**). These results suggest that *gdp1* and *oli2* upregulate the expression levels of *ARF3, KAN2*, and *YAB5*, and slightly enhance leaf abaxialization of *as2*. However, the effects of *gdp1-1* and *oli2-1* on leaf abaxialization were much weaker than those of *rpl4d-3* (**Figures 8A,B**). One possible explanation for this observation is that a ribosome biosynthesis defect has the potential to induce leaf abaxialization in *as2-1*, but at the same time, GDP1 and OLI2 also play roles in the promotion of leaf abaxialization. To examine this possibility, we generated *as2-1 rpl4d-3 gdp1-1* and *as2 rpl4d-3 oli2-1*. These triple mutants did not show alleviation of the leaf polarity defect of *as2-1 rpl4d-3*, but had more severe developmental defects judging from their smaller shoot size and formation of filamentous first and/or second leaves (**Figure 8D**). This result suggests that *GDP1* and *OLI2* are dispensable for leaf polarity establishment and/or maintenance even in the *as2* background.

## Discussion

### Involvement of GDP1 and OLI2 in Ribosome Biogenesis

In this study, we found that *GDP1* and *OLI2* are strongly expressed in developing tissues and encode different nucleolar proteins. Their loss-of-function mutations cause developmental phenotypes that are often observed in RBF-defective mutants and affect rRNA processing. These results suggest that the two nucleolar proteins are involved in ribosome biogenesis.

OLI2 is a homolog of yeast Nop2, which has methyltransferase activity and modifies 25S rRNA at a specific cytosine residue (m5C2870; Sharma et al., 2013). This modification is evolutionarily conserved at an equivalent site in animals (Maden, 1988) and plants (Burgess et al., 2015). However, m5C2870 is dispensable in yeast as catalytically inactive Nop2 is able to complement a *nop2* deletion mutant (Bourgeois et al., 2015). Nop2 is a component of the LSU processome (McCann et al., 2015) and acts as one of the B factors necessary to carry out the processing of 27S rRNA into 25S and 5.8S rRNAs (Hong et al., 1997; Talkish et al., 2012). Depletion of Nop2 results in increased accumulation of 27S rRNA and corresponding decreases in levels of mature 25S and 5.8S rRNAs (Hong et al., 1997).

The conserved m5C methylation site in 25S rRNA in plants indicates the presence of a functional ortholog of Nop2 in Arabidopsis. However, this modification normally presents in *oli2* mutant alleles (named *nop2a* in Burgess et al., 2015). This may be due to functional redundancy among the *OLI2* gene family; double mutants between *nop2a* and *nop2b* are lethal (Burgess et al., 2015). Therefore, direct evidence for OLI2 as an m5C methyltransferase is lacking at present. In addition, while both *nop2* in yeast and *oli2* show overaccumulation of precursors that retain the intact ITS2, and *nop2* reduces the 25S rRNA level, *oli2* does not detectably reduce the 25S rRNA level (**Figure 7C**). This suggests that the overall processing mechanisms involving OLI2 and Nop2 are conserved in eukaryotes, but their details may differ. Taken together, these results indicate that OLI2 plays a role as an RBF; however, whether it is a genuine Nop2 ortholog remains to be determined.

In this study we also identified a novel nucleolar RBF named GDP1, which contains a G-patch domain. In yeast, two G-patch domain proteins, Gno1 and Pfa1, are involved in ribosome biogenesis (Guglielmi and Werner, 2002; Lebaron et al., 2005; Chen et al., 2014). According to PANTHER version 12.0^2^ (Mi et al., 2016), GDP1 was classified as a protein family (PTHR23149) that also contains Gno1 and its ortholog in human, PINX1. These three proteins harbor a G-patch domain in their amino-terminal side. On the other hand, Pfa1 has a G-patch domain in its carboxy-terminal side and also has an R3H domain. Gno1 associates with both 90S pre-ribosomes and early pre-60S ribosomes, but only transiently with early pre-40S ribosomes (Chen et al., 2014). The lack of Gno1 leads to processing defects within the 5′-ETS and ITS1 (Chen et al., 2014). Downstream rRNA intermediates, such as 27S rRNAs and mature 18S and 25S rRNAs are reduced in *gno1*. In *gdp1*, 35S and 32S rRNAs increased, but in contrast to *gno1*, 27SA/B rRNAs increased without detectably affecting the steady-state levels of 25S and 18S rRNAs (**Figure 7C**). Thus, *gdp1*, like *gno1*, seems to affect multiple steps of rRNA processing, but it has a partially different effect on rRNA processing. Thus, whether GDP1 has an orthologous function to Gno1 remains to be elucidated. An emerging molecular role of G-patch domain protein is the activation of DEAH/RHA helicases (Robert-Paganin et al., 2015). Both Gno1 and Pfa1 function in ribosome biogenesis in close association with the DEAH/RHA helicase, Prp43. Future characterization of an Arabidopsis homolog of Prp43 may provide additional insight into the function of GDP1.

### Relationships between R-Proteins and RBFs

In a series of genetic crosses, we found an additive or synergistic genetic interaction depending on the combination of single mutants examined. The combination between *gdp1* and most r-protein mutants examined resulted in an additive phenotype. This suggests that processes of GDP1-dependent ribosome biogenesis and those involving these r-proteins occur largely independently. On the other hand, the synergistic genetic interaction between *gdp1* and *oli2* suggests the interdependence of GDP1 and OLI2 functions in ribosome biogenesis. In yeast, Gno1 acts at multiple steps of ribosome biogenesis in close association with Prp43 (Lebaron et al., 2005, 2009; Chen et al., 2014). Prp43 is expected to function in rearrangement of pre-ribosomal particles through its RNA helicase activity (Lebaron et al., 2005, 2009). Nop2 is an essential protein and is required for hierarchical recruitment of the B factors (Hong et al., 1997; Talkish et al., 2012). Thus, successive changes in the geometry of pre-ribosomes are important for the progression of subsequent processes. The interdependence of GDP1 and OLI2 functions may have arisen from the dynamic nature of ribosome biogenesis.

Unexpectedly, *gdp1 oli2* did not show marked increases in the levels of rRNA intermediates compared to its parental single mutants (**Figure 7**). This discrepancy may have been due to the nature of the experiments, in that our RNA analyses determined the steady-state levels of rRNA intermediates. Further characterization of GDP1 and OLI2 and measurement of rRNA processing rate could resolve this issue.

Another notable interaction was found between *gdp1* and *rps6a*. Despite being a component of the 40S subunit, RPS6A was shown to interact with the histone deacetylase HD2B and NUCLEOSOME ASSEMBLY PROTEIN1 (NAP1), and to regulate transcription of 45S rRNA (Kim et al., 2014; Son et al., 2015). Thus, the exceptionally strong interaction of *rps6a* with *gdp1* among other r-protein mutants may have been due to the role of RPS6A in ribosome biogenesis. Similar synergistic relationships were also reported between another pair of RBF mutants, *apum23* and *nuc-l1* (Abbasi et al., 2010). In the same work, double mutants between *apum23* and *as1/2 enhancer5* (*ae5*)/*rpl28a* or *ae6*/*rpl5a* were reported not to show more severe phenotypes than the respective single mutants (Abbasi et al., 2010). As *ae6* is an allele of *oli5*, and *gdp1 oli5* shows an additive phenotype in relation to the cell proliferation defect (**Figure 3**), evaluation of genetic interactions between ribosome-related mutants based solely on shoot size could miss a more fundamental interaction. In summary, our genetic experiments suggested that a combination of weak defects in RBFs may cause a marked reduction of the flow of ribosome biogenesis, resulting in a strong cell proliferation defect.

### Genetic Defects in RBFs Do Not Always Induce Strong Leaf Abaxialization in *as2*

Several reports described enhanced leaf abaxialization in *as2* by r-protein mutants (Byrne, 2009; Horiguchi et al., 2012; Machida et al., 2015). Recently, mutations in three RBF genes, *NUC-L1, RH10*, and *RID2*, were also shown to strongly enhance leaf abaxialization in *as2* (Matsumura et al., 2016). In yeast, orthologs of these three proteins are components of, or RBFs associated with, the SSU processome. These findings suggested a tight link between the SSU processome and AS2-dependent cell fate decision (Matsumura et al., 2016). In addition, *APUM23* encodes a homolog of yeast Nop9, and *apum23* also strongly enhances the *as2* leaf polarity phenotype (Huang et al., 2014). Although the precise functions of APUM23 and Nop9 could differ, APUM23 is able to partially complement the *nop9* mutant phenotype in yeast (Huang et al., 2014). Interestingly, Nop9 is also a component of the SSU processome (Zhang et al., 2016). In contrast to reports regarding these RBFs (Huang et al., 2014; Matsumura et al., 2016), *gdp1, oli2*, and even *gdp1 oli2* had little or very mild effects on the enhancement of leaf abaxialization in the *as2* background (**Figure 8**). These results suggest that not all of the RBFs are tightly linked with leaf adaxial/abaxial polarity. Gno1 was found in the SSU processome, pre-40S, and pre-60S ribosomes (Chen et al., 2014). If GDP1 is also a component of the SSU processome, this argues against a link between the SSU processome and leaf abaxial/adaxial polarity regulation. A possible explanation for this discrepancy is that there may be a key structure or a subcomplex within the SSU processome, dysfunction of which is linked to leaf abaxialization in *as2*. This is a likely scenario, as the SSU processome is a 2.2-MDa ribonucleoprotein composed of modular subcomplexes (Pérez-Fernández et al., 2007). On the other hand, Nop2 is a component of the LSU processome in yeast, and OLI2 is a putative ortholog of Nop2. Several scenarios may explain the limited effect of *oli2* on leaf abaxialization. First, the LSU processome may not have a link to the regulation of adaxial/abaxial polarity. However, we consider this to be unlikely, because we reported previously that mutations in *RPL4D* encoding a 60S r-protein have a very strong effect on leaf abaxialization in *as2* (Horiguchi et al., 2011), and, in yeast, RPL4 is incorporated during an early stage of pre-60S formation (Gamalinda et al., 2014; Stelter et al., 2015; Chen et al., 2017). Similar to the SSU processome, the LSU processome may also have a link to adaxial/abaxial patterning through its local structures. As ribosome biogenesis is a highly dynamic process, surveillance of ribosome biogenesis defects at every step is probably impossible. Consistently, in yeast cells with specific genetic backgrounds, aberrant ribosomal subunits can escape from the surveillance systems and engage in translation (Rodríguez-Galán et al., 2015). On the other hand, surveillance at multiple key checkpoints to sense local defects of nascent ribosomes represents an easier strategy. The differential contributions of RBFs to leaf abaxial/adaxial polarity regulation may be correlated with their relative importance to such checkpoints.

Our genetic analyses on *gdp1* and *oli2* illustrated that a genetic defect in ribosome biogenesis leads to cell proliferation defect but it does not have a strong effect to enhance leaf abaxialization when it is introduced into the *as2* background. A contrasting example is found in *rpl4d* where *rpl4d* single mutants do not have a clear cell proliferation defect yet it causes very strong leaf abaxialization in the *as2* background (Horiguchi et al., 2011). These examples suggest there may be a critical point during ribosome biogenesis that is linked to the regulation of leaf adaxial/abaxial polarity. It is noteworthy that *oli2* has a statistically significant effect to upregulate *YAB5* expression when compared to *as2* (**Figure 8C**) but it did not result in strong leaf abaxialization. In *as2 rh10*, strong leaf abaxialization is dependent on *ARF3* (Matsumura et al., 2016). The expression levels of *ARF3* in *as2 rh10* and *as2 gdp1 oli2* were four and threefold higher than that in the wild type plants, respectively (Matsumura et al., 2016; **Figure 8C**). Although plant samples used in these studies were different (shoot tips or whole shoots), the differential sensitivities of leaf abaxialization in these mutants might be attributable to the degree of enhanced *ARF3* expression. An increasing number of characterized ribosome biogenesis mutants of Arabidopsis should offer an opportunity to localize such a point through quantitative and comparative analyses of these mutants in a future study.

A critical issue concerning the above discussion is whether there is actually such a checkpoint in plants. In mammals, ribosome biogenesis defects lead to activation of the tumor suppressor, p53, which acts as a transcriptional activator and triggers stress responses, such as cell cycle arrest, senescence, and apoptosis (Kastenhuber and Lowe, 2017). Under normal conditions, p53 is ubiquitinated by the E3 ubiquitin ligase, MDM2, and subjected to proteolysis. On the other hand, ribosome biogenesis defects inhibit MDM2 activity through direct interaction between the 5S rRNA-RPL5-RPL11 complex and MDM2 (Sun et al., 2010; Sloan et al., 2013; Bursac et al., 2014). However, plants have orthologs of neither p53 nor MDM2. Recently, Ohbayashi et al. (2017) showed that the NAC transcription factor gene, *SRIW1*, is upregulated in *rid2*. Notably, *rid2 sriw1* still suffers from the rRNA processing defects, but the developmental phenotypes, such as impaired callus formation and pointed leaf formation, were suppressed (Ohbayashi et al., 2017). These results indicated that the observed developmental phenotypes are indirect consequences of rRNA processing defects, and are more directly mediated by the ribosomal stress response in which SRIW1 plays a central role, similar to p53 in animals.

In this study, we showed that *gdp1 oli2* double mutants had a very strong cell proliferation defect, but enhanced the leaf polarity defect of *as2* less strongly. How a defect in a general cellular process such as ribosome biogenesis interferes with a specific developmental process is an important emerging issue in plant developmental and cell biology (Tsukaya et al., 2013). We suggest that the cell proliferation defect in leaf primordia and leaf abaxialization triggered by a mutation in RBF or an r-protein gene could be mediated by different mechanisms. It will be interesting to examine whether these developmental phenotypes are mediated by SRIW1.

## Author Contributions

GH and HT designed and conducted the experiments. KK, JT, HC, KF, and GH performed the experiments. GH and HT wrote the manuscript.

## Conflict of Interest Statement

The authors declare that the research was conducted in the absence of any commercial or financial relationships that could be construed as a potential conflict of interest. The handling Editor declared a past co-authorship with several of the authors HT, GH.

## Footnotes

1 https://imagej.nih.gov/ij/

2 http://www.pantherdb.org/publications.jsp

## References

[B1] Abbasi N. Kim H. B. Park N. I. Kim H. S. Kim Y. K. Park Y. I. (2010). APUM23, a nucleolar Puf domain protein, is involved in pre-ribosomal RNA processing and normal growth patterning in Arabidopsis. *Plant J.* 64 960–976. 10.1111/j.1365-313X.2010.04393.x 21143677

[B2] Aravind L. Koonin E. V. (1999). G-patch: a new conserved domain in eukaryotic RNA-processing proteins and type D retroviral polyproteins. *Trends Biochem. Sci.* 24 342–344. 10.1016/S0968-0004(99)01437-1 10470032

[B3] Bourgeois G. Ney M. Gaspar I. Aigueperse C. Schaefer M. Kellner S. (2015). Eukaryotic rRNA modification by yeast 5-methylcytosine-methyltransferases and human proliferation-associated antigen p120. *PLOS ONE* 10:e133321. 10.1371/journal.pone.0133321 26196125 PMC4510066

[B4] Brown J. W. S. Shaw P. J. Shaw P. Marshall D. F. (2005). *Arabidopsis* nucleolar protein database (AtNoPDB). *Nucleic Acids Res.* 33 D633–D636. 10.1093/nar/gki052 15608277 PMC540006

[B5] Burgess A. L. David R. Searle I. R. (2015). Conservation of tRNA and rRNA 5-methylcytosine in the kingdom Plantae. *BMC Plant Biol.* 15:119. 10.1186/s12870-015-0580-8 26268215 PMC4535395

[B6] Bursac S. Brdovcak M. C. Donati G. Volarevic S. (2014). Activation of the tumor suppressor p53 upon impairment of ribosome biogenesis. *Biochim. Biophys. Acta* 1842 817–830. 10.1016/j.bbadis.2013.08.014 24514102

[B7] Byrne M. E. (2009). A role for the ribosome in development. *Trends Plant Sci.* 14 512–519. 10.1016/j.tplants.2009.06.009 19716746

[B8] Casanova-Sáez R. Candela H. Micol J. L. (2014). Combined haploinsufficiency and purifying selection drive retention of *RPL36a* paralogs in Arabidopsis. *Sci. Rep.* 4:4122. 10.1038/srep04122 24535089 PMC3927210

[B9] Chaker-Margot M. Barandun J. Hunziker M. Klinge S. (2017). Architecture of the yeast small subunit processome. *Science* 355:eaal1880. 10.1126/science.aal1880 27980088

[B10] Chen W. Xie Z. Yang F. Ye K. (2017). Stepwise assembly of the earliest precursors of large ribosomal subunits in yeast. *Nucleic Acids Res.* 45 6837–6847. 10.1093/nar/gkx254 28402444 PMC5499802

[B11] Chen Y. L. Capeyrou R. Humbert O. Mouffok S. Kadri Y. A. Lebaron S. (2014). The telomerase inhibitor Gno1p/PINX1 activates the helicase Prp43p during ribosome biogenesis. *Nucleic Acids Res.* 42 7330–7345. 10.1093/nar/gku357 24823796 PMC4066782

[B12] Cho H. K. Ahn C. S. Lee H. S. Kim J. K. Pai H. S. (2013). Pescadillo plays an essential role in plant cell growth and survival by modulating ribosome biogenesis. *Plant J.* 76 393–405. 10.1111/tpj.12302 23909681

[B13] Clough S. J. Bent A. F. (1998). Floral dip: a simplified method for Agrobacterium-mediated transformation of *Arabidopsis thaliana*. *Plant J.* 16 735–743. 10.1046/j.1365-313x.1998.00343.x 10069079

[B14] Comella P. Pontvianne F. Lahmy S. Vignols F. Barbezier N. DeBures A. (2007). Characterization of a ribonuclease III-like protein required for cleavage of the pre-rRNA in the 3’ETS in *Arabidopsis*. *Nucleic Acids Res.* 36 1163–1175. 10.1093/nar/gkm1130 18158302 PMC2275086

[B15] Donnelly P. M. Bonetta D. Tsukaya H. Dengler R. E. Dengler N. G. (1999). Cell cycling and cell enlargement in developing leaves of *Arabidopsis*. *Dev. Biol.* 215 407–419. 10.1006/dbio.1999.9443 10545247

[B16] Droll D. Archer S. Fenn K. Delhi P. Matthews K. Clayton C. (2010). The trypanosome Pumilio-domain protein PUF7 associates with a nuclear cyclophilin and is involved in ribosomal RNA maturation. *FEBS Lett.* 584 1156–1162. 10.1016/j.febslet.2010.02.018 20153321 PMC2855960

[B17] Ebersberger I. Simm S. Leisegang M. S. Schmitzberger P. Mirus O. von Haeseler A. (2013). The evolution of the ribosome biogenesis pathway from a yeast perspective. *Nucleic Acids Res.* 42 1509–1523. 10.1093/nar/gkt1137 24234440 PMC3919561

[B18] Fernández-Pevida A. Kressler D. de la Cruz J. (2015). Processing of preribosomal RNA in *Saccharomyces cerevisiae*. *Wiley Interdiscip. Rev. RNA* 6 191–209. 10.1002/wrna.1267 25327757

[B19] Fujikura U. Horiguchi G. Ponce M. R. Micol J. L. Tsukaya H. (2009). Coordination of cell proliferation and cell expansion mediated by ribosome-related processes in the leaves of *Arabidopsis thaliana*. *Plant J.* 59 499–508. 10.1111/j.1365-313X.2009.03886.x 19392710

[B20] Gamalinda M. Ohmayer U. Jakovljevic J. Kumcuoglu B. Woolford J. Mbom B. (2014). A hierarchical model for assembly of eukaryotic 60S ribosomal subunit domains. *Genes Dev.* 28 198–210. 10.1101/gad.228825.113 24449272 PMC3909792

[B21] Grandi P. Rybin V. Bassler J. Petfalski E. Strauss D. Marzioch M. (2002). 90S pre-ribosomes include the 35S pre-rRNA, the U3 snoRNP, and 40S subunit processing factors but predominantly lack 60S synthesis factors. *Mol. Cell* 10 105–115. 10.1016/S1097-2765(02)00579-8 12150911

[B22] Griffith M. E. Mayer U. Capron A. Ngo Q. A. Surendrarao A. McClinton R. (2007). The *TORMOZ* gene encodes a nucleolar protein required for regulated division planes and embryo development in *Arabidopsis*. *Plant Cell* 19 2246–2263. 10.1105/tpc.106.042697 17616738 PMC1955705

[B23] Guglielmi B. Werner M. (2002). The yeast homolog of human PinX1 is involved in rRNA and small nucleolar RNA maturation, not in telomere elongation inhibition. *J. Biol. Chem.* 277 35712–35719. 10.1074/jbc.M205526200 12107183

[B24] Hang R. Liu C. Ahmad A. Zhang Y. Lu F. Cao X. (2014). *Arabidopsis* protein arginine methyltransferase 3 is required for ribosome biogenesis by affecting precursor ribosomal RNA processing. *Proc. Natl. Acad. Sci. U.S.A.* 111 16190–16195. 10.1073/pnas.1412697111 25352672 PMC4234621

[B25] Hasegawa J. Sakamoto Y. Nakagami S. Aida M. Sawa S. Matsunaga S. (2016). Three-dimensional imaging of plant organs using a simple and rapid transparency technique. *Plant Cell Physiol.* 57 462–472. 10.1093/pcp/pcw027 26928932

[B26] Henras A. K. Plisson-Chastang C. O’Donohue M.-F. Chakraborty A. Gleizes P. E. (2015). An overview of pre-ribosomal RNA processing in eukaryotes. *Wiley Interdiscip. Rev. RNA* 6 225–242. 10.1002/wrna.1269 25346433 PMC4361047

[B27] Hisanaga T. Kawade K. Tsukaya H. (2015). Compensation: a key to clarifying the organ-level regulation of lateral organ size in plants. *J. Exp. Bot.* 66 1055–1063. 10.1093/jxb/erv028 25635111

[B28] Hong B. Brockenbrough J. S. Wu P. Aris J. P. (1997). Nop2p is required for pre-rRNA processing and 60S ribosome subunit synthesis in yeast. *Mol. Cell. Biol.* 17 378–388. 10.1128/MCB.17.1.378 8972218 PMC231762

[B29] Horiguchi G. Ferjani A. Fujikura U. Tsukaya H. (2006a). Coordination of cell proliferation and cell expansion in the control of leaf size in *Arabidopsis thaliana*. *J. Plant Res.* 119 37–42. 10.1007/s10265-005-0232-4 16284709

[B30] Horiguchi G. Fujikura U. Ferjani A. Ishikawa N. Tsukaya H. (2006b). Large-scale histological analysis of leaf mutants using two simple leaf observation methods: identification of novel genetic pathways governing the size and shape of leaves. *Plant J.* 48 638–644. 10.1111/j.1365-313X.2006.02896.x 17076802

[B31] Horiguchi G. Kim G. T. Tsukaya H. (2005). The transcription factor AtGRF5 and the transcription coactivator AN3 regulate cell proliferation in leaf primordia of *Arabidopsis thaliana*. *Plant J.* 43 68–78. 10.1111/j.1365-313X.2005.02429.x 15960617

[B32] Horiguchi G. Mollá-Morales A. Pérez-Pérez J. M. Kojima K. Robles P. Ponce M. R. (2011). Differential contributions of ribosomal protein genes to *Arabidopsis thaliana* leaf development. *Plant J.* 65 724–736. 10.1111/j.1365-313X.2010.04457.x 21251100

[B33] Horiguchi G. Tsukaya H. (2011). Organ size regulation in plants: insights from compensation. *Front. Plant Sci.* 2:24. 10.3389/fpls.2011.00024 22639585 PMC3355714

[B34] Horiguchi G. Van Lijsebettens M. Candela H. Micol J. L. Tsukaya H. (2012). Ribosomes and translation in plant developmental control. *Plant Sci.* 19 24–34. 10.1016/j.plantsci.2012.04.008 22682562

[B35] Huang C. K. Huang L. F. Huang J. J. Wu S. J. Yeh C. H. Lu C. A. (2010). A DEAD-box protein, AtRH36, is essential for female gametophyte development and is involved in rRNA biogenesis in Arabidopsis. *Plant Cell Physiol.* 51 694–706. 10.1093/pcp/pcq045 20378763

[B36] Huang T. Kerstetter R. A. Irish V. F. (2014). APUM23, a PUF family protein, functions in leaf development and organ polarity in *Arabidopsis*. *J. Exp. Bot.* 65 1181–1191. 10.1093/jxb/ert478 24449383 PMC3935572

[B37] Iwakawa H. Iwasaki M. Kojima S. Ueno Y. Soma T. Tanaka H. (2007). Expression of the *ASYMMETRIC LEAVES2* gene in the adaxial domain of Arabidopsis leaves represses cell proliferation in this domain and is critical for the development of properly expanded leaves. *Plant J.* 51 173–184. 10.1111/j.1365-313X.2007.03132.x 17559509

[B38] Kastenhuber E. R. Lowe S. W. (2017). Putting p53 in context. *Cell* 170 1062–1078. 10.1016/j.cell.2017.08.028 28886379 PMC5743327

[B39] Kim Y.-K. Kim S. Shin Y. Hur Y.-S. Kim W.-Y. Lee M.-S. (2014). Ribosomal Protein S6, a target of rapamycin, is involved in the regulation of rRNA genes by possible epigenetic changes in *Arabidopsis*. *J. Biol. Chem.* 289 3901–3912. 10.1074/jbc.M113.515015 24302738 PMC3924259

[B40] Konikkat S. Woolford J. L. Jr. (2017). Principles of 60S ribosomal subunit assembly emerging from recent studies in yeast. *Biochem. J.* 474 195–214. 10.1042/BCJ20160516 28062837 PMC5555582

[B41] Kornprobst M. Turk M. Kellner N. Cheng J. Flemming D. Kos-Braun I. (2016). Architecture of the 90S pre-ribosome: a structural view on the birth of the eukaryotic ribosome. *Cell* 166 380–393. 10.1016/j.cell.2016.06.014 27419870

[B42] Kumakura N. Otsuki H. Tsuzuki M. Takeda A. Watanabe Y. (2013). *Arabidopsis* AtRRP44A is the functional homolog of Rrp44/Dis3 an exosome component, is essential for viability and is required for RNA processing and degradation. *PLOS ONE* 8:e79219. 10.1371/journal.pone.0079219 24244451 PMC3820695

[B43] Lange H. Holec S. Cognat V. Pieuchot L. Le Ret M. Canaday J. (2008). Degradation of a polyadenylated rRNA maturation by-product involves one of the three RRP6-like proteins in *Arabidopsis thaliana*. *Mol. Cell. Biol.* 28 3038–3044. 10.1128/MCB.02064-07 18285452 PMC2293077

[B44] Lange H. Sement F. M. Gagliardi D. (2011). MTR4, a putative RNA helicase and exosome co-factor, is required for proper rRNA biogenesis and development in *Arabidopsis thaliana*. *Plant J.* 68 51–63. 10.1111/j.1365-313X.2011.04675.x 21682783

[B45] Layat E. Sáez-Vásquez J. Tourmente S. (2012). Regulation of Pol I-transcribed 45S rDNA and Pol III-transcribed 5S rDNA in Arabidopsis. *Plant Cell Physiol.* 53 267–276. 10.1093/pcp/pcr177 22173098

[B46] Lebaron S. Froment C. Fromont-Racine M. Rain J. Monsarrat B. Caizergues-Ferrer M. (2005). The splicing ATPase Prp43 is a component of multiple preribosomal particles. *Mol. Cell. Biol.* 25 9269–9282. 10.1128/MCB.25.21.9269-9282.2005 16227579 PMC1265834

[B47] Lebaron S. Papin C. Capeyrou R. Chen Y.-L. Froment C. Monsarrat B. (2009). The ATPase and helicase activities of Prp43p are stimulated by the G-patch protein Pfa1p during yeast ribosome biogenesis. *EMBO J.* 28 3808–3819. 10.1038/emboj.2009.335 19927118 PMC2797057

[B48] Létoquart J. Huvelle E. Wacheul L. Bourgeois G. Zorbas C. Graille M. (2014). Structural and functional studies of Bud23-Trm112 reveal 18S rRNA N7-G1575 methylation occurs on late 40S precursor ribosomes. *Proc. Natl. Acad. Sci. U.S.A.* 111 E5518–E5526. 10.1073/pnas.1413089111 25489090 PMC4280632

[B49] Li N. Yuan L. Liu N. Shi D. Li X. Tang Z. (2009). *SLOW WALKER2*, a NOC1/MAK21 homologue, is essential for coordinated cell cycle progression during female gametophyte development in Arabidopsis. *Plant Physiol.* 151 1486–1497. 10.1104/pp.109.142414 19734265 PMC2773048

[B50] Liu M. Shi D. Q. Yuan L. Liu J. Yang W. C. (2010). *SLOW WALKER3*, encoding a putative DEAD-box RNA helicase, is essential for female gametogenesis in *Arabidopsis*. *J. Integr. Plant Biol.* 52 817–828. 10.1111/j.1744-7909.2010.00972.x 20738726

[B51] Machida C. Nakagawa A. Kojima S. Takahashi H. Machida Y. (2015). The complex of ASYMMETRIC LEAVES (AS) proteins plays a central role in antagonistic interactions of gene s for leaf polarity specification in *Arabidopsis*. *Wiley Interdiscip. Rev. Dev. Biol.* 4 655–671. 10.1002/wdev.196 26108442 PMC4744985

[B52] Maden B. E. (1988). Locations of methyl groups in 28S rRNA of *Xenopus laevis* and man. Clustering in the conserved core of molecule. *J. Mol. Biol.* 201 289–314. 10.1016/0022-2836(88)90139-8 3418702

[B53] Matsumura Y. Ohbayashi I. Takahashi H. Kojima S. Ishibashi N. Keta S. (2016). A genetic link between epigenetic repressor AS1-AS2 and a putative small subunit processome in leaf polarity establishment of *Arabidopsis*. *Biol. Open* 5 942–954. 10.1242/bio.019109 27334696 PMC4958277

[B54] McCann K. L. Charette J. M. Vincent N. G. Baserga S. J. (2015). A protein interaction map of the LSU processome. *Genes Dev.* 29 862–875. 10.1101/gad.256370.114 25877921 PMC4403261

[B55] Mi H. Huang X. Muruganujan A. Tang H. Mills C. Kang D. (2016). PANTHER version 11: expanded annotation data from gene ontology and reactome pathways, and data analysis tool enhancements. *Nucleic Acids Res.* 45 D183–D189. 10.1093/nar/gkw1138 27899595 PMC5210595

[B56] Missbach S. Weis B. L. Martin R. Simm S. Bohnsack M. T. Schleiff E. (2013). 40S ribosome biogenesis co-factors are essential for gametophyte and embryo development. *PLOS ONE* 8:e54084. 10.1371/journal.pone.0054084 23382868 PMC3559688

[B57] Nakagawa T. Suzuki T. Murata S. Nakamura S. Hino T. Maeo K. (2007). Improved Gateway binary vectors: high-performance vectors for creation of fusion constructs in transgenic analysis of plants. *Biosci. Biotechnol. Biochem.* 71 2095–2100. 10.1271/bbb.70216 17690442

[B58] Nerurkar P. Altvater M. Gerhardy S. Schütz S. Fischer U. Weirich C. (2015). Eukaryotic ribosome assembly and nuclear export. *Int. Rev. Cell Mol. Biol.* 319 107–140. 10.1016/bs.ircmb.2015.07.002 26404467

[B59] O’Day C. L. Chavanikamannil F. Abelson J. (1996). 18S rRNA processing requires the RNA helicase-like protein Rrp3. *Nucleic Acids Res.* 24 3201–3207. 10.1093/nar/24.16.3201 8774901 PMC146083

[B60] Ohbayashi I. Konishi M. Ebine K. Sugiyama M. (2011). Genetic identification of Arabidopsis RID2 as an essential factor involved in pre-rRNA processing. *Plant J.* 67 49–60. 10.1111/j.1365-313X.2011.04574.x 21401745

[B61] Ohbayashi I. Lin C. Y. Shinohara N. Matsumura Y. Machida Y. Horiguchi G. (2017). Evidence for a role of ANAC082 as a ribosomal stress response mediator leading to growth defects and developmental alterations in Arabidopsis. *Plant Cell* 29 2644–2660. 10.1105/tpc.17.00255 28899981 PMC5774571

[B62] Palm D. Simm S. Darm K. Weis B. L. Ruprecht M. Schleiff E. (2016). Proteome distribution between nucleoplasm and nucleolus and its relation to ribosome biogenesis in *Arabidopsis thaliana*. *RNA Biol.* 13 441–454. 10.1080/15476286.2016.1154252 26980300 PMC5038169

[B63] Pendle A. F. Clark G. P. Boon R. Lewandowska D. Lam Y. W. Andersen J. (2005). Proteomic analysis of the *Arabidopsis* nucleolus suggests novel nucleolar functions. *Mol. Biol. Cell* 16 260–269. 10.1091/mbc.E04-09-0791 15496452 PMC539170

[B64] Pérez-Fernández J. Román Á De Las Rivas J. Bustelo X. R. Dosil M. (2007). The 90S preribosome is a multimodular structure that is assembled through a hierarchical mechanism. *Mol. Cell. Biol.* 27 5414–5429. 10.1128/MCB.00380-07 17515605 PMC1952102

[B65] Petricka J. J. Nelson T. M. (2007). Arabidopsis nucleolin affects plant development and patterning. *Plant Physiol.* 144 173–186. 10.1104/pp.106.093575 17369435 PMC1913809

[B66] Phipps K. R. Charette J. Baserga S. J. (2011). The small subunit processome in ribosome biogenesis – progress and prospects. *Wiley Interdiscip. Rev. RNA* 2 1–21. 10.1002/wrna.57 21318072 PMC3035417

[B67] Pinon V. Etchells J. P. Rossignol P. Collier S. A. Arroyo J. M. Martienssen R. A. (2008). Three *PIGGYBACK* genes that specifically influence leaf patterning encode ribosomal proteins. *Development* 135 1315–1324. 10.1242/dev.016469 18305008

[B68] Robert-Paganin J. Réty S. Leulliot N. (2015). Regulation of DEAH/RHA helicases by G-patch proteins. *Biomed. Res. Int.* 2015:931857. 10.1155/2015/931857 25692149 PMC4322301

[B69] Rodríguez-Galán O. García-Gómez J. J. Kressler D. de la Cruz J. (2015). Immature large ribosomal subunits containing the 7S pre-rRNA can engage in translation in *Saccharomyces cerevisiae*. *RNA Biol.* 12 838–846. 10.1080/15476286.2015.1058477 26151772 PMC4615593

[B70] Sáez-Vasquez J. Caparros-Ruiz D. Barneche F. Echeverría M. (2004). A plant snoRNP complex containing snoRNAs, Fibrillarin, and Nucleolin-like proteins is competent for both rRNA gene binding and pre-rRNA processing in vitro. *Mol. Cell. Biol.* 24 7284–7297. 10.1128/MCB.24.16.7284-7297.2004 15282326 PMC479724

[B71] Sardana R. White J. P. Johnson A. W. (2013). The rRNA methyltransferase Bud23 shows functional interaction with components of the SSU processome and RNase MRP. *RNA* 19 828–840. 10.1261/rna.037671.112 23604635 PMC3683916

[B72] Sharma S. Yang J. Watzinger P. Kötter P. Entian K. D. (2013). Yeast Nop2 and Rcm1 methylate C2870 and C2278 of the 25S rRNA, respectively. *Nucleic Acids Res.* 41 9062–9076. 10.1093/nar/gkt679 23913415 PMC3799443

[B73] Shi D. Q. Liu J. Xiang Y. H. Ye D. Sundaresan V. Yang W. C. (2005). *SLOW WALKER1*, essential for gametogenesis in Arabidopsis, encodes a WD40 protein involved in 18S ribosomal RNA biogenesis. *Plant Cell* 17 2340–2354. 10.1105/tpc.105.033563 15980260 PMC1182493

[B74] Sloan K. E. Bohnsack M. T. Watkins N. J. (2013). The 5S RNP couples p53 homeostasis to ribosome biogenesis and nucleolar stress. *Cell Rep.* 5 237–247. 10.1016/j.celrep.2013.08.049 24120868 PMC3808153

[B75] Son O. Kim S. Shin Y. J. Kim W. Y. Koh H. Cheon C. I. (2015). Identification of nucleosome assembly protein 1 (NAP1) as an interacting partner of plant ribosomal protein S6 (RPS6) and a positive regulator of rDNA transcription. *Biochem. Biophys. Res. Commun.* 465 200–205. 10.1016/j.bbrc.2015.07.150 26241676

[B76] Stelter P. Huber F. M. Kunze R. Flemming D. Hoelz A. Hurt E. (2015). Coordinated ribosomal L4 protein assembly into the pre-ribosome is regulated by its eukaryote-specific extension. *Mol. Cell* 58 854–862. 10.1016/j.molcel.2015.03.029 25936803 PMC6742479

[B77] Sun Q. Zhu X. Qi J. An W. Lan P. Tan D. (2017). Molecular architecture of the 90S small subunit pre-ribosome. *eLife* 6:e22086. 10.7554/eLife.22086 28244370 PMC5354517

[B78] Sun X. X. Wang Y. G. Xirodimas D. P. Dai M. S. (2010). Perturbation of 60 S ribosomal biogenesis results in ribosomal protein L5- and L11-dependent p53 activation. *J. Biol. Chem.* 285 25812–25821. 10.1074/jbc.M109.098442 20554519 PMC2919143

[B79] Szakonyi D. Byrne M. E. (2011). Ribosomal protein L27a is required for growth and patterning in *Arabidopsis thaliana*. *Plant J.* 65 269–281. 10.1111/j.1365-313X.2010.04422.x 21223391

[B80] Tafforeau L. Zorbas C. Langhendries J. L. Mullineux S. T. Stamatopoulou V. Mullier R. (2013). The complexity of human ribosome biogenesis revealed by systematic nucleolar screening of pre-rRNA processing factors. *Mol. Cell* 22 539–551. 10.1016/j.molcel.2013.08.011 23973377

[B81] Talkish J. Zhang J. Jakovljevic J. Horsey E. W. Woolford J. L. Jr. (2012). Hierarchical recruitment into nascent ribosomes of assembly factors required for 27SB pre-rRNA processing in *Saccharomyces cerevisiae*. *Nucleic Acids Res.* 40 8646–8661. 10.1093/nar/gks609 22735702 PMC3458554

[B82] Thomson E. Rappsilber J. Tollervey D. (2007). Nop9 is an RNA binding protein present in pre-40S ribosomes and required for 18S rRNA synthesis in yeast. *RNA* 13 2165–2174. 10.1261/rna.747607 17956976 PMC2080597

[B83] Tsukaya H. Byrne M. E. Horiguchi G. Sugiyama M. Van Lijsebettens M. Lenhard M. (2013). How do ‘housekeeping’ genes control organogenesis? –unexpected new findings on the role of housekeeping genes in cell and organ differentiation. *J. Plant Res.* 126 3–15. 10.1007/s10265-012-0518-2 22922868

[B84] Turner A. J. Knox A. A. Prieto J. L. McStay B. Watkins N. J. (2009). A novel small-subunit processome assembly intermediate that contains the U3 snoRNP, Nucleolin, RRP5, and DBP4. *Mol. Cell. Biol.* 29 3007–3017. 10.1128/MCB.00029-09 19332556 PMC2682003

[B85] Waites R. Hudson A. (1995). *phantastica*: a gene required for dorsoventrality of leaves in *Antirrhinum majus*. *Development* 121 2143–2154.

[B86] Warner J. R. (1999). The economics of ribosome biosynthesis in yeast. *Trends Biochem. Sci.* 24 437–440. 10.1016/S0968-0004(99)01460-7 10542411

[B87] Weis B. L. Kovacevic J. Missbach S. Schleiff E. (2015a). Plant-specific features of ribosome biogenesis. *Trends Plant Sci.* 20 729–740. 10.1016/j.tplants.2015.07.003 26459664

[B88] Weis B. L. Palm D. Missbach S. Bohnsack M. T. Schleiff E. (2015b). atBRX1-1 and atBRX1-2 are involved in an alternative rRNA processing pathway in *Arabidopsis thaliana*. *RNA* 21 415–425. 10.1261/rna.047563.114 25605960 PMC4338337

[B89] Weis B. L. Missbach S. Marzi J. Bohnsack M. T. Schleiff E. (2014). The 60S associated ribosome biogenesis factor LSG1-2 is required for 40S maturation in *Arabidopsis thaliana*. *Plant J.* 80 1043–1056. 10.1111/tpj.12703 25319368

[B90] Wieckowski Y. Schiefelbein J. (2012). Nuclear ribosome biogenesis mediated by the DIM1A rRNA dimethylase is required for organized root growth and epidermal patterning in *Arabidopsis*. *Plant Cell* 24 2839–2856. 10.1105/tpc.112.101022 22829145 PMC3426118

[B91] Winter D. Vinegar B. Nahal H. Ammar R. Wilson G. V. Provart N. J. (2007). An “electronic fluorescent pictograph” browser for exploring and analyzing large-scale biological data set. *PLOS ONE* 2:e718. 10.1371/journal.pone.0000718 17684564 PMC1934936

[B92] Yao Y. Ling Q. Wang H. Huang H. (2008). Ribosomal proteins promote leaf adaxial identity. *Development* 135 1325–1334. 10.1242/dev.017913 18305007

[B93] Zakrzewska-Placzek M. Souret F. F. Sobczyk G. J. Green P. J. Kufel J. (2010). *Arabidopsis thaliana* XRN2 is required for primary cleavage in the pre-ribosomal RNA. *Nucleic Acids Res.* 38 4487–4502. 10.1093/nar/gkq172 20338880 PMC2910052

[B94] Zhang C. Muench D. G. (2015). A nucleolar PUF RNA-binding protein with specificity for a unique RNA sequence. *J. Biol. Chem.* 290 30108–30118. 10.1074/jbc.M115.691675 26487722 PMC4706012

[B95] Zhang J. Harnpicharnchai P. Jakovljevic J. Tang L. Guo Y. Oeffinger M. (2007). Assembly factors Rpf2 and Rrs1 recruit 5S rRNA and ribosomal proteins rpL5 and rpL11 into nascent ribosomes. *Genes Dev.* 21 2580–2592. 10.1101/gad.1569307 17938242 PMC2000323

[B96] Zhang J. McCann K. L. Qiu C. Gonzalez L. E. Baserga S. J. Hall T. M. T. (2016). Nop9 is a PUF-like protein that prevents premature cleavage to correctly process pre-18S rRNA. *Nat. Commun.* 11:13085. 10.1038/ncomms13085 27725644 PMC5062617

