# Supplementary material for: Two Nucleolar Proteins, GDP1 and OLI2, Function As Ribosome Biogenesis Factors and Are Preferentially Involved in Promotion of Leaf Cell Proliferation without Strongly Affecting Leaf Adaxial–Abaxial Patterning in Arabidopsis thaliana
Source: Front Plant Sci. 2018 Jan 9;8:2240. doi: 10.3389/fpls.2017.02240 (PMC5767255; doi:10.3389/fpls.2017.02240)
Supplement: Supplementary file 2 [file Presentation_1.PPTX]

## Slide 1
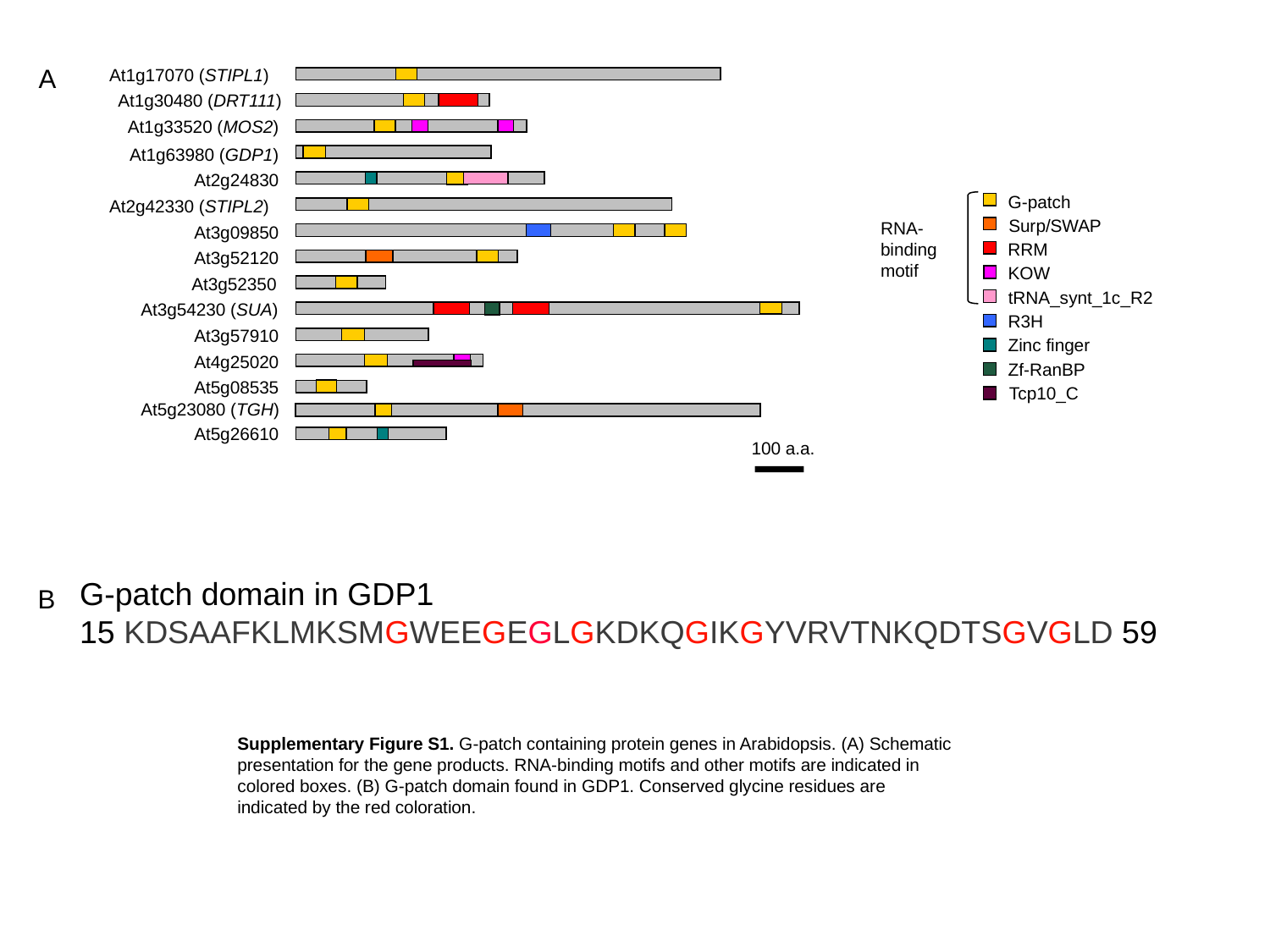

A
At1g17070 (STIPL1)
At1g30480 (DRT111)
At1g33520 (MOS2)
At1g63980 (GDP1)
At2g24830
G-patch
Surp/SWAP
RNA-binding motif
RRM
KOW
tRNA_synt_1c_R2
R3H
Zinc finger
Zf-RanBP
Tcp10_C
At2g42330 (STIPL2)
At3g09850
At3g52120
At3g52350
At3g54230 (SUA)
At3g57910
At4g25020
At5g08535
At5g23080 (TGH)
At5g26610
100 a.a.
G-patch domain in GDP1
15 KDSAAFKLMKSMGWEEGEGLGKDKQGIKGYVRVTNKQDTSGVGLD 59
B
Supplementary Figure S1. G-patch containing protein genes in Arabidopsis. (A) Schematic presentation for the gene products. RNA-binding motifs and other motifs are indicated in colored boxes. (B) G-patch domain found in GDP1. Conserved glycine residues are indicated by the red coloration.

## Slide 2
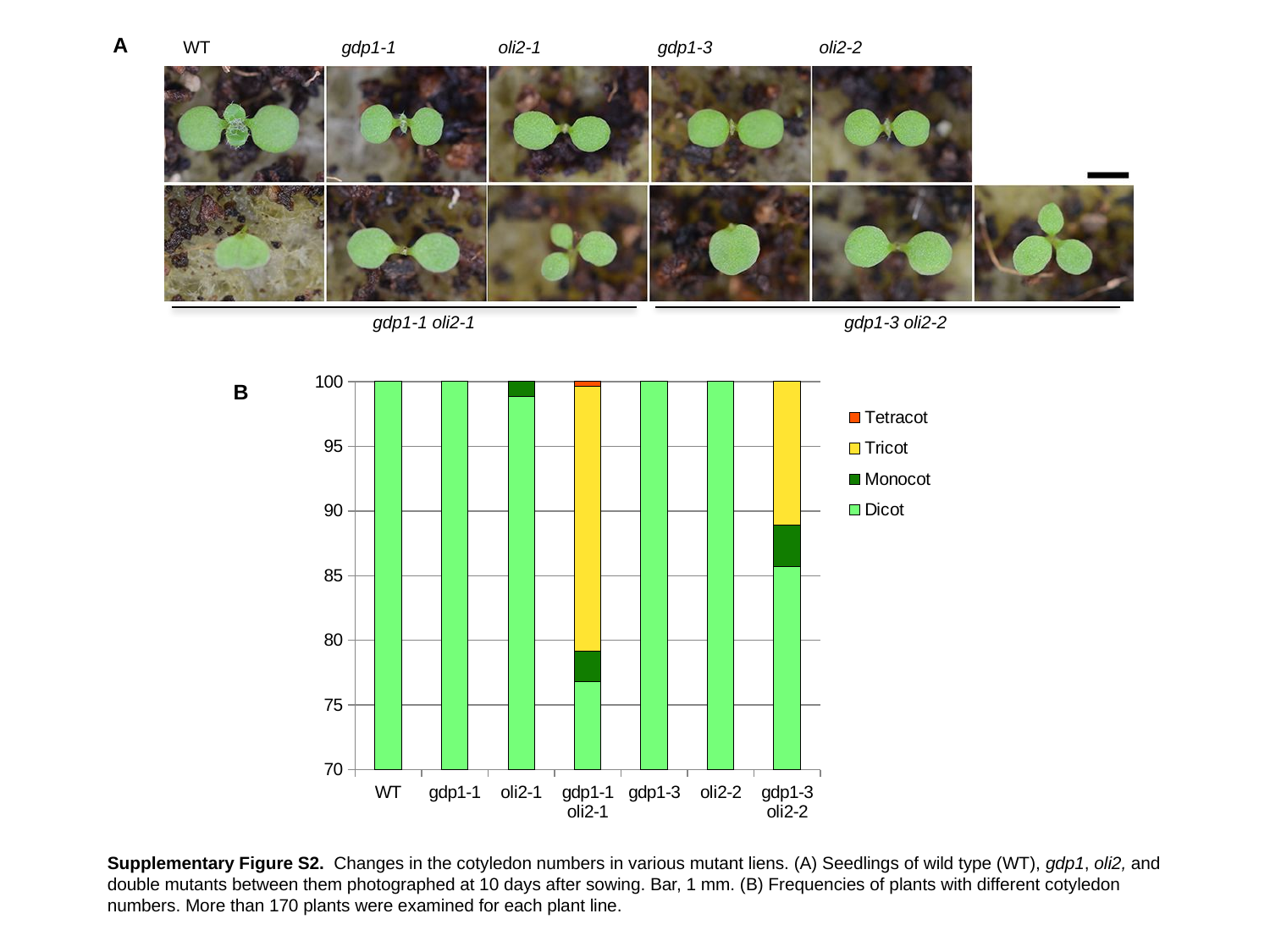

A
WT gdp1-1 oli2-1 gdp1-3 oli2-2
gdp1-1 oli2-1 gdp1-3 oli2-2
### Chart
| Category | Dicot | Monocot | Tricot | Tetracot |
|---|---|---|---|---|
| WT | 100.0 | 0.0 | 0.0 | 0.0 |
| gdp1-1 | 100.0 | 0.0 | 0.0 | 0.0 |
| oli2-1 | 98.87640449438203 | 1.123595505617978 | 0.0 | 0.0 |
| gdp1-1 oli2-1 | 76.79180887372014 | 2.389078498293515 | 20.4778156996587 | 0.341296928327645 |
| gdp1-3 | 100.0 | 0.0 | 0.0 | 0.0 |
| oli2-2 | 100.0 | 0.0 | 0.0 | 0.0 |
| gdp1-3 oli2-2 | 85.67567567567568 | 3.243243243243243 | 11.08108108108108 | 0.0 |B
Supplementary Figure S2. Changes in the cotyledon numbers in various mutant liens. (A) Seedlings of wild type (WT), gdp1, oli2, and double mutants between them photographed at 10 days after sowing. Bar, 1 mm. (B) Frequencies of plants with different cotyledon numbers. More than 170 plants were examined for each plant line.
